# Level set-based XFEM modelling of the multi-scale hygro-mechanical behaviour of oak wood using morphological input from $\mu$CT

**DOI:** 10.1007/s00466-025-02618-0

**Published:** 2025-03-29

**Authors:** M. A. Livani, A. S. J. Suiker, M. P. F. H. L. van Maris, E. Bosco

**Affiliations:** 1 Department of the Built Environment, Eindhoven University of Technology, P.O. Box 513, 5600 MB Eindhoven, The Netherlands; 2 Department of Mechanical Engineering, Eindhoven University of Technology, Eindhoven, The Netherlands

**Keywords:** Micro-computed tomography, Level set-based image segmentation, Extended finite element method, Asymptotic homogenization, Oak anatomy

## Abstract

A computational multi-scale model is presented to predict the macroscopic hygro-mechanical behaviour of oak wood, based on detailed three-dimensional mesoscopic representations of entire oak growth rings obtained by X-ray micro-computed tomography ($\mu$CT). The 3D meso-structural volumes acquired by $\mu$CT scanning consist of arrays of voxels, with the grayscale intensity values of the voxels denoting the local material densities. A level set-based image segmentation method is applied to distinguish the individual meso-structural phases, including the cell walls and voids (lumen and vessels). A dedicated algorithm based on the spatial gradient of the level set function accurately identifies the local material directions in the cell walls. The individual phases in the meso-scale cellular structure are discretized using the extended finite element method. Here, a moment fitting scheme is applied for an efficient numerical integration in the elements intersected by cell wall boundaries. Finally, asymptotic homogenization is used for computing the effective macro-scale response of oak wood from the hygro-mechanical response of the underlying meso-structure. The macro-scale hygro-mechanical behaviour calculated by the multi-scale model for oak growth rings agrees well with experimental values from the literature. Further, the meso-scale response computed for oak growth rings subjected to a representative moisture content variation allows to identify local, critical sites in which mesoscopic hygro-mechanical damage may occur. The effective hygro-mechanical properties calculated by the multi-scale model may serve as an input for predicting the moisture-dependent mechanical response of oak wood structures and objects subjected to arbitrary hygro-mechanical loading paths.

## Introduction

Oak wood is a versatile material that is widely employed across a broad range of applications, including buildings and civil engineering structures [10, 22] and artistic pieces, such as sculptures, decorated cabinets and panel paintings [44, 46, 73]. However, the sensitivity of oak wood to moisture variations can lead to significant deformations of oak objects and structures, and consequently to damage development. For example, relative humidity fluctuations in museums, often caused by the entering public, can cause tensile stresses in susceptible oak art objects that promote fracture, such as the through-thickness cracks observed in cabinet door panels and the transverse cracks and delaminations observed in the decorative layers of panel paintings [4, 7, 8, 23, 29, 45, 46, 70]. In order to limit and/or prevent climate-induced damage in oak objects and structures, a thorough understanding of the hygro-mechanical response of oak wood is necessary, which requires a detailed study of the characteristics of its complex multi-scale behaviour [65].

**Fig. 1:**
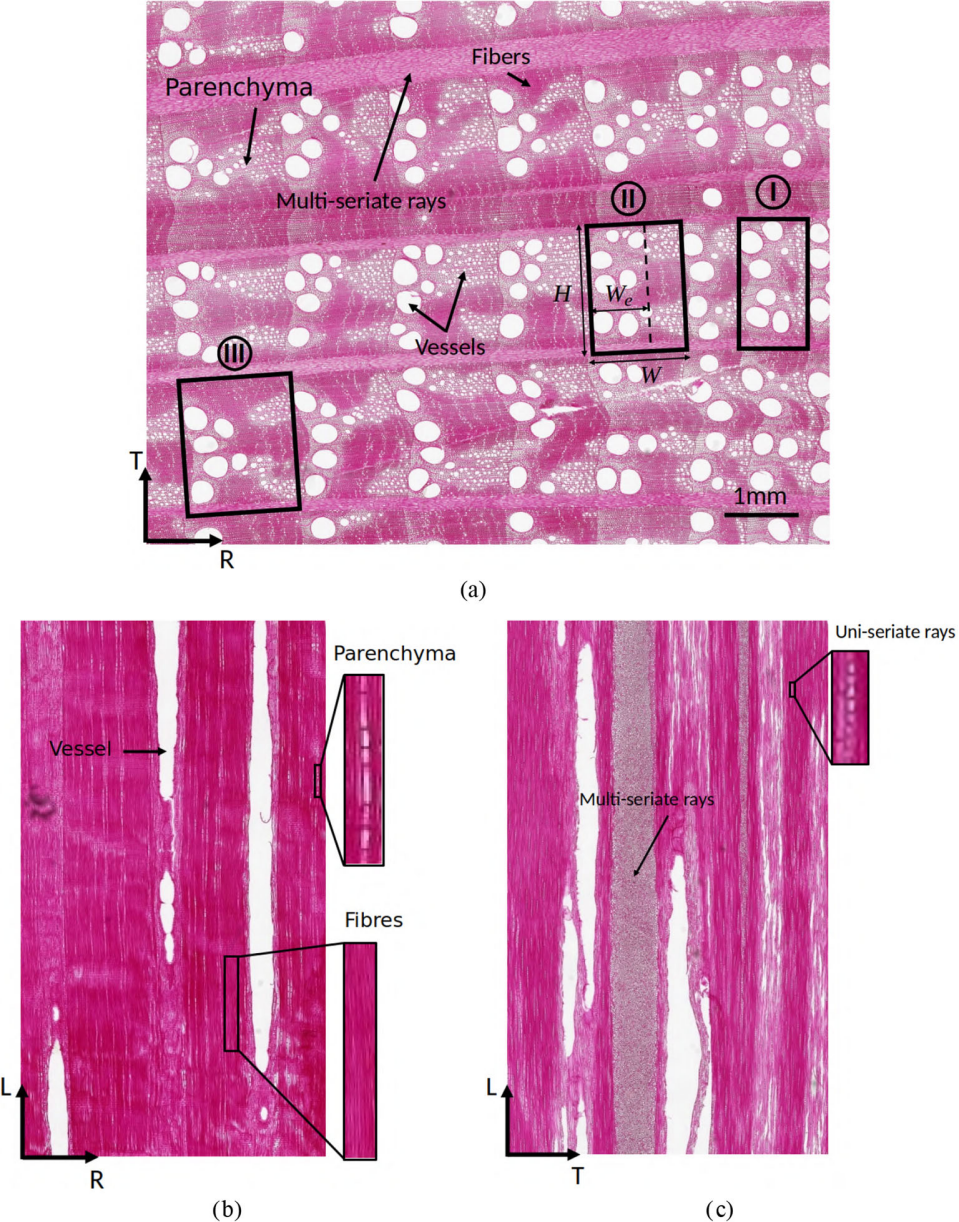
Microscopy images of oak sections taken along the three principal material planes. **a** Section along the *transverse* (R–T) plane. The cellular structure of the growth rings reveals a pattern of large earlywood vessels and much smaller latewood vessels. The three rectangular regions of width *W* and height *H* indicated by the black boxes I, II, and III refer to different growth rings. For growth ring II, the transition between the earlywood and latewood regions is indicated by a dashed line, with their widths equal to $W_e$ and ($W-W_e$), respectively. **b** Section along the *radial* (R–L) plane. The dark and light regions indicate fibers and parenchyma cells, respectively. The elongated oval-shaped voids represent the vessels. **c** Section along the *tangential* (T–L) plane. The specific cross-sections of multi-seriate and uni-seriate rays can be identified. The figure has been reprinted from [43]

At the macroscopic scale, oak wood can be considered an orthotropic solid, exhibiting distinct properties along its three principal material directions, i.e., the longitudinal (L) direction of the tree stem, and the radial (R) and tangential (T) directions of the seasonal growth rings that appear as concentric layers in the cross-section of the stem. At the mesoscopic scale, oak wood is characterized by a cellular structure comprising various cell types, which are depicted in the microscopy images in Fig. 1a–c that respectively consider sections along the transverse (R-T) plane, radial (R-L) plane, and tangential (T-L) plane. These cell types include libriform fibers and fiber tracheids, which are elongated hollow tubes primarily aligned along the longitudinal direction and serve to provide mechanical resistance. In addition, axial parenchyma cells can be identified, which have a shorter length and a thinner cell wall than fibers and are predominantly located between vessels. Vessels are tubular structures oriented in the longitudinal direction of oak wood and have a larger diameter and a shorter length than fibers. The cellular structure further contains uni-seriate and multi-seriate ray parenchyma cells, or rays, which are prism-shaped and oriented radially within growth rings. Figure 1a also displays three different growth rings I, II, and III, with their width *W* and height *H* indicated by the black boxes. For growth ring II, the transition between the earlywood and latewood regions is designated by a dashed line, with their widths equal to $W_e$ and ($W-W_e$), respectively. The various cell types described above clearly exhibit a different spatial organization across the three growth rings, where earlywood regions typically contain fiber tracheids, axial parenchyma and large vessels, while latewood comprises libriform fibers, axial parenchyma, and small vessels. More details on the cellular structure of oak wood can be found in [20, 36].

In the literature, several multi-scale models have been proposed for predicting the effective mechanical and hygroscopic properties of wood based on its underlying morphological features. For the analysis of softwoods, such as spruce, the approximately regular cellular structure of fiber tracheids and axial parenchyma is mimicked through a periodic configuration of honeycomb cells, and the effective macroscale material response is determined from two-dimensional [40, 59, 63, 68] or three-dimensional [61, 64, 66] homogenization procedures. For the analysis of the more complex multi-scale behaviour of hardwoods, such as oak, the contributions of ray parenchyma cells and vessels also need to be accounted for, which is often done through adopting somewhat idealized meso-structural configurations in the homogenization approach [17, 32, 40, 49, 50]. To improve this aspect, in [2, 58] representative image-based two-dimensional geometries of oak wood growth rings are used to calculate the effective mechanical properties in the transverse (R-T) anatomical plane of oak. Furthermore, Livani et al. [43] recently proposed a three-dimensional multi-scale model of oak wood, in which the hygro-mechanical behaviour at four different scales of observation (i.e., the nano-, micro-, meso- and macro-scales) is stepwisely coupled by means of asymptotic homogenization and Voigt averaging techniques. At the meso-scale, the relatively complex three-dimensional cellular structure is constructed by extruding detailed two-dimensional microscopy images of growth rings along the third, longitudinal direction. This model provides accurate predictions of the effective three-dimensional hygro-mechanical response of oak, which are consistent with experimental observations. However, the idealized representation of the meso-structure in the longitudinal direction resulting from the extrusion procedure prevents it from reliably capturing local strain and stress fields in the radial and tangential planes.

To significantly advance the understanding of the multi-scale response of oak wood, it is thus essential to use high-fidelity three-dimensional representations of its complex cellular meso-structure. This work proposes a novel computational approach for predicting the hygro-mechanical response of oak wood by integrating accurate, three-dimensional morphologies of entire oak growth rings obtained by X-ray micro-computer tomography ($\mu$CT) into a multi-scale framework based on asymptotic homogenization. As demonstrated in [9, 37, 57], $\mu$CT scanning is very suitable for visualizing the interior features of wood meso-structures and obtaining high-quality digital information on their 3D geometries and properties. In this study, a two-scale (meso- to macro-scale) version of the model proposed in [43] is developed. The two-scale model employs asymptotic homogenization to determine the effective macro-scale response of oak wood from the response of the underlying meso-structure. Following [3], the solution of the equilibrium problem is written as an asymptotic expansion. This leads to a set of boundary value problems at the meso-scale referred to as the *cell problems*. The solution to the cell problems provides the meso-scale fluctuations of the displacement field, which are subsequently used to compute the effective macro-scale material properties and the local stress and strain fields generated in the considered meso-structural domains under applied macroscopic hygro-mechanical loading conditions. Note that the specific hygro-mechanical loading considered in this study is relatively low in magnitude, as it reflects typical conditions in museum environments. This justifies the use of the classic asymptotic homogenization method, which focuses on linear problems under the assumptions of small strains and linear material behavior.

The 3D meso-structural volume obtained by $\mu$CT scanning consists of an array of voxels, with the grayscale intensity values of the voxels denoting the local material densities. A level set-based image segmentation method is applied to these data to distinguish the individual meso-structural phases composing the oak wood, i.e., the cell walls and the voids (represented by lumen and vessels). The response of the cell walls is characterized by a hygro-elastic constitutive behaviour, defined by moisture-dependent elastic coefficients and hygro-expansion coefficients in the orthotropic L- R- and T-directions [43]. A dedicated algorithm based on the spatial gradient of the level set function is developed to accurately identify the L- R- and T-directions in the local material points of the cell walls. For solving the cell problems defined on the meso-scale cellular structure, the structure is discretized using the extended finite element method [24]. Employing the partition of unity concept, the enriched shape functions used in the extended finite element method (XFEM) allow to describe phase boundaries that run arbitrarily through the underlying finite element discretization, see [28, 67, 76] for various applications. This enables to accurately model the interfaces between the cell walls and the voids with a regular, non-conforming mesh. Since the locations of the phase boundaries are already defined by the level set function used in the image segmentation method, the level set function can be straightforwardly applied as the governing variable in the Heaviside enrichment function to capture the locations of the phase boundaries in the XFEM approach. Despite the efficiency of the level set-based XFEM approach, the solution of the cell problems over the relatively large three-dimensional oak domains considered involves a significant computational burden. To reduce the computational time, a moment fitting scheme [33, 52] is applied for the efficient numerical integration of the hygro-mechanical response in the elements intersected by cell wall boundaries. In conclusion, with the incorporation of realistic 3D meso-structures, the approach presented in this paper enables accurate predictions of effective macro-scale properties while also allowing for detailed local analyses of strain and stress distributions across all anatomical planes. This advancement provides a deeper understanding of how hygro-mechanical mechanisms contribute to meso-scale degradation in oak objects. Furthermore, the modelling of fully 3D oak morphologies enables direct comparisons with experimental tests on the same samples, providing a basis for future integrated numerical-experimental studies.

The paper is organized as follows. The multi-scale model of oak wood is described in Sect. 2. Section 3 presents the meso-scale unit cells obtained by combining X-ray $\mu$CT data with a level set-based image segmentation procedure, followed by a description of the hygro-mechanical properties of the cell walls. Section 4 contains the XFEM discretization of the oak wood meso-structure and summarizes the moment-fitting numerical integration scheme. Section 5 presents the moisture-dependent hygro-elastic properties of oak computed at the macro-scale with the asymptotic homogenization procedure, together with the reconstructed meso-scale stress and strain fields. Finally, in Sect. 6 the main conclusions of the study are summarized.

Throughout the paper the following notation for vectors, tensors, and tensor products is employed: $a, \textbf{a}, \textbf{A}$ and $^n \! \textbf{A}$ denote, respectively, a scalar, a vector, a second-order tensor, and an *n*th-order tensor. By making use of Einstein’s summation convention, vector and tensor operations are defined as follows: a dyadic product is represented by $\textbf{a} \otimes \textbf{b} = a_i b_j ~\textbf{e}_i \otimes \textbf{e}_j$, and inner products are defined as $\textbf{A}\cdot \textbf{b} = A_{ij}b_j ~\textbf{e}_i$, $\textbf{A}\cdot \textbf{B} = A_{ij}B_{jk}\textbf{e}_i~ \otimes \textbf{e}_k$, $\textbf{A}:\textbf{B} = A_{ij}B_{ij}$ and , with $\textbf{e}_i$ ($i = x, y, z$) the unit vectors of a Cartesian basis. The superscript T is used to indicate the transpose of matrices and vectors. The symbols $\varvec{\nabla }$ and $\varvec{\nabla }\cdot$ respectively denote the gradient and the divergence operators, as defined by $\varvec{\nabla } f = \partial f / \partial x \ \textbf{e}_x + \partial f / \partial y \ \textbf{e}_y + \partial f / \partial z \ \textbf{e}_z$ and $\varvec{\nabla } \cdot \textbf{f} = \partial f_x / \partial x + \partial f_y / \partial y + \partial f_z / \partial z$.

**Fig. 2:**
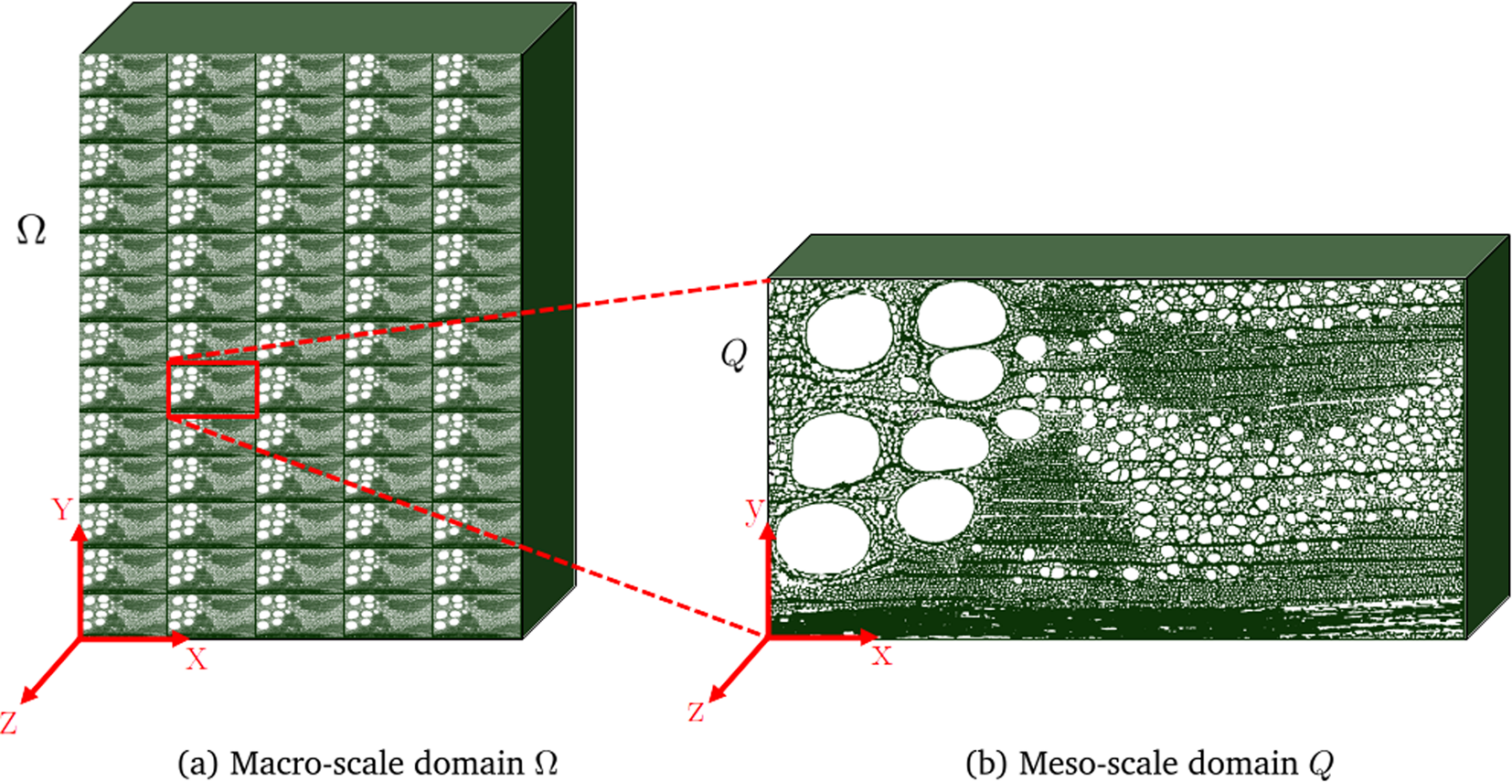
Spatial domains for the asymptotic homogenization procedure. **a** Macro-scale domain $\Omega$ with a characteristic length $\mathcal {L}$ and **b** underlying periodic meso-scale unit cell *Q* with a characteristic length *L*

## Multi-scale methodology

The effective hygro-mechanical properties of oak wood are determined via an asymptotic homogenization procedure [1, 3, 21, 55, 56]. Asymptotic homogenization allows to represent the intrinsically heterogeneous oak wood material as an equivalent homogeneous medium, for which the effective properties are extracted from the meso-scale response through an averaging procedure based on rigorous mathematical principles. The applied homogenization framework for a heterogeneous material subjected to hygro-mechanical conditions is detailed in [5, 6] and is briefly summarized below.

Figure 2a shows a macro-scale material (oak wood) with a heterogeneous meso-scale structure. The three-dimensional macro-scale domain $\Omega$ is constructed as a *periodic* repetition of the meso-scale unit cell *Q* depicted in Fig. 2b. Denoting the characteristic lengths of the macro- and meso-scale domains as $\mathcal {L}$ and $L=\eta \mathcal {L}$, it is assumed that a strong separation between the scales holds, such that $\eta \ll 1$. Further, the displacement, stress and strain fields governing the hygro-mechanical behaviour are supposed to vary smoothly at the macro-scale, while they are considered to be periodic at the meso-scale. Consequently, these fields may be assumed to explicitly depend on two variables: a slow variable $\textbf{X}$ at the macro-scale level and a fast variable $\textbf{x}=\textbf{X}/\eta$ at the meso-scale level. Further, in the absence of body forces, the mechanical equilibrium equation reads

1 $$\begin{aligned} \varvec{\nabla } \cdot \varvec{\sigma } = \textbf{0} \hspace{2pt}, \end{aligned}$$

where $\varvec{\sigma }$ is the Cauchy stress tensor that satisfies the following hygro-elastic constitutive relation:

2 $$\begin{aligned} \varvec{\sigma }(\textbf{X})=  ^{4}{\mathbf {{C}}}(\textbf{x}): \left( {\varvec{\varepsilon }(\textbf{X})}- \mathcal { \varvec{\beta }}(\textbf{x}) \Delta m(\textbf{X}) \right) \hspace{2pt}. \end{aligned}$$

Here, the strain tensor $\varvec{\varepsilon }(\textbf{X})$ is the symmetric part of the gradient of the displacement field $\textbf{u} (\textbf{X})$, i.e., $\varvec{\varepsilon }(\textbf{X}) = \textrm{sym}\left( \varvec{\nabla } \textbf{u} (\textbf{X}) \right)$. Moreover, $\Delta m(\textbf{X})$ is a prescribed macroscopic moisture content variation, defined as the difference between the current moisture content *m* and a reference moisture content $m_0$. The material properties at the underlying meso-scale level determine the macro-scale response. They are expressed by local constitutive tensors that are piecewise smooth periodic functions of the meso-scale variable $\textbf{x}$, i.e., the elastic stiffness tensor $^4\textbf{C}={^4\textbf{C}(\textbf{x})}$ and the hygroscopic expansion tensor $\varvec{\beta } = \varvec{\beta }(\textbf{x})$ per $\%$ of moisture content change. The local elastic stiffness tensor $^4\textbf{C}(\textbf{x})$ satisfies the standard major and minor symmetry conditions, $C_{ijkl} = C_{jikl} = C_{ijlk} = C_{klij}$, and is required to be positive-definite. The local hygroscopic expansion tensor $\mathcal { \varvec{\beta }}(\textbf{x})$ is postulated to be symmetric, with $\beta _{ij}=\beta _{ii}\delta _{ij}$ and $\delta _{ij}$ the Kronecker delta symbol, as hygroscopic strains primarily induce expansion or contraction along the principal material directions. Although not indicated in equation (2), the tensors $^4\textbf{C}$ and $\varvec{\beta }$ depend on the current value of the moisture content *m*, which thus is assumed to be uniform at the mesoscopic scale.

Asymptotic homogenization is based on formulating the displacement field as an asymptotic expansion in terms of $\eta$ as

3 $$\begin{aligned} \textbf{u}(\textbf{X})=\textbf{u}_0(\textbf{X},\textbf{X}/\eta ) +\eta \textbf{u}_1(\textbf{X},\textbf{X}/\eta )+\eta ^2 \textbf{u}_2 (\textbf{X},\textbf{X}/\eta ) + \mathcal {O} (\eta ^3) \, , \end{aligned}$$

where the Landau symbol $\mathcal {O}(.)$ denotes the order of magnitude of the corresponding term. The effective material response of the equivalent homogeneous medium is recovered from the meso-structural level through an averaging procedure that departs from inserting the constitutive law (2) into the equilibrium equation (1), followed by substituting the asymptotic expansion of the displacement field (3) in the resulting expression. This leads to the formulation of two sets of mathematical problems, one defined at the *meso-scale* and one at the *macro-scale*.

- *Meso-scale problem*. The mathematical problem at the meso-scale focuses on the calculation of the influence functions $^{3}\textbf{N}_{1}$ and $\textbf{b}_{1}$. The influence functions $^{3}\textbf{N}_{1}$ and $\textbf{b}_{1}$ describe the periodic meso-scale fluctuations of the displacement field of the cellular structure of oak in response to variations of the average macroscopic strain and moisture content, respectively. The functions can be calculated as the solutions of so-called *cell problems*, which are represented by two boundary value problems for the unit cell *Q*. The cell problems only depend on the meso-structural constitutive properties $^{4}\textbf{C}(\textbf{x})$ and $\varvec{\beta }(\textbf{x})$ and have the form
  4 $$\begin{aligned} \begin{array}{llr} \varvec{\nabla }\cdot \left(  ^{4}\textbf{C}(\textbf{x}):(\varvec{\nabla } ^{3}\textbf{N}_{1}\left( \textbf{x})+ ^{4}\textbf{I}^{S} \right) \right) &  = &   ^{3}\varvec{0} ~, \\ \varvec{\nabla }\cdot \left(  ^{4}\textbf{C}(\textbf{x}): \left( \varvec{\nabla } \textbf{b}_{1}(\textbf{x})-\varvec{\beta }(\textbf{x}) \right) \right) &  = &  \varvec{0} ~, \end{array} \end{aligned}$$
  where $^{4}\textbf{I}^{S}$ is the fourth-order symmetric identity tensor, defined as $I^S_{ijkl} = (\delta _{il}\delta _{jk} + \delta _{ik}\delta _{jl} )/2$. The cell problems in equations (4)$_{1,2}$ are completed by *periodic boundary conditions* for the unknown influence functions $^3\textbf{N}_{1}$ and $\textbf{b}_1$. Periodic boundary conditions require that the values assumed by each influence function at corresponding points on opposite boundaries (left and right, top and down, front and back) are equal. Further, to warrant the uniqueness of the solution, the influence functions are prescribed to vanish on average across the unit cell.
  The cell problems in equations (4)$_{1,2}$ are solved for a given meso-structural geometry utilizing the level set-based XFEM approach, which will be discussed in Sect. 4. Subsequently, the computed influence functions $^3\textbf{N}_{1}$ and $\textbf{b}_1$ are used to construct the local stress and strain fields in the oak meso-structural domain. For this purpose, the local displacement field $\textbf{u}_1$ in expression (3) is written as the sum of a macroscopic contribution $\textbf{u}_0$, defined in relation (3), and a mesoscopic contribution $\textbf{v}_1(\textbf{x})$, i.e.,
  5 $$\begin{aligned} \textbf{u}_1(\textbf{x}) = \textbf{u}_0 + \textbf{v}_1(\textbf{x}) = \textbf{u}_0 + ~^3\textbf{N}_{1}:\varvec{\nabla }\textbf{u}_0 + \textbf{b}_1\Delta m ~, \end{aligned}$$
  where, for notational convenience, the dependency of $\textbf{u}_0$ and $\Delta m$ on the slow variable $\textbf{X}$ has been omitted. Note from expression (5) that the mesoscopic displacement $\textbf{v}_1$ is obtained as the sum of two contributions, which respectively are related to the mechanical influence function $~^3\textbf{N}_{1}$ and the hygroscopic influence function $\textbf{b}_1$.
  Consider now two hygro-mechanical loading cases, where the oak meso-structure is subjected to a macroscopic moisture content variation $\Delta m$ under *i)* *constrained expansion* conditions with zero average deformation, and *ii)* *free expansion* conditions with zero average stress. In the first case the macroscopic strain is given by $\bar{\varvec{\varepsilon }}_0= \textrm{sym}\left( \varvec{\nabla }\textbf{u}_0 \right) = \varvec{0}$, while in the second case the macroscopic strain equals $\bar{\varvec{\varepsilon }}_0= \textrm{sym}\left( \varvec{\nabla }\textbf{u}_0\right) = \bar{\varvec{\beta }} \Delta m$, where $\bar{\varvec{\beta }}$ is the effective hygro-expansion coefficient computed from the influence functions through equation (8), as explained below. The local strain $\varvec{\varepsilon }(\textbf{x})$ at the mesoscopic level can be calculated by taking the spatial gradient of equation (5) and combining the result with the definitions for the macroscopic strain $\bar{\varvec{\varepsilon }}_0$ given above, which leads to
  6 $$\begin{aligned} \varvec{\varepsilon }(\textbf{x})  &   = \textrm{sym}\left( \varvec{\nabla }\left( \textbf{u}_0 + \textbf{v}_1(\textbf{x}) \right) \right) \nonumber \\  &   = \left\{ \begin{array}{ll} \varvec{\nabla }\textbf{b}_1 \Delta m \,\,\,\,\,\,\,\,\,\,\,\,\,\,\,\,\,\,\,\,\,\,\,\,\,\,\,\,\,\,\,\,\,\,\,\,\,\,\,\,\,\,\,\,\,\,\,\,\,\, \mathrm {for~constrained~expansion,} \\ \left( \bar{\varvec{\beta }} {+} \varvec{\nabla }(~^3\textbf{N}_{1}):\bar{\varvec{\beta }} {+} \varvec{\nabla }\textbf{b}_1 \right) \Delta m\, \mathrm {for~free~expansion} .\end{array} \right. \nonumber \\ \end{aligned}$$
  Subsequenty, the local, moisture-induced stress field $\varvec{\sigma }(\textbf{x})$ in the meso-structure is computed by inserting equation (6) in the mesoscopic hygro-mechanical constitutive relation
  7 $$\begin{aligned} \varvec{\sigma }(\textbf{x})= ~^4\textbf{C}(\textbf{x}): \left( \varvec{\varepsilon }(\textbf{x}) - \varvec{\beta }(\textbf{x}) \Delta m\right) ~. \end{aligned}$$
  The material parameters characterizing the mesoscopic elastic stiffness tensor $^4\textbf{C}(\textbf{x})$ and the hygro-expansion tensor $\varvec{\beta }(\textbf{x})$ will be discussed in Sect. 3.2.
- *Macro-scale problem*. After the influence functions $^{3}\textbf{N}_{1}$ and $\textbf{b}_{1}$ are determined from the solution of equations (4)$_{1,2}$, the effective elastic tensor $^{4}\bar{\textbf{C}}$ and hygroscopic expansion tensor $\bar{\varvec{\beta }}$ can be computed as
  8 $$\begin{aligned} \begin{array}{rcl}  ^{4}\bar{\textbf{C}} & =&  \displaystyle {\dfrac{1}{|Q|}\int _{Q} ^4\textbf{C}(\textbf{x}):(\varvec{\nabla } ^{3}\textbf{N}_{1}(\textbf{x})+ ^{4}\textbf{I}^{S}) \, \textrm{d}Q} ~, \\ \bar{\varvec{\beta }} & =&  \displaystyle { \dfrac{1}{|Q|} ^{4}\bar{\textbf{C}}^{-1}:\int _{Q} ^4\textbf{C}(\textbf{x}):(\varvec{\beta }(\textbf{x})-\varvec{\nabla } \textbf{b}_{1}(\textbf{x})) \, \textrm{d}Q} ~. \end{array} \end{aligned}$$
  Further details on the solution of the above cell problems with the level set-based XFEM approach are provided in Sect. 4.

## Meso-scale model of oak wood

### Geometry

#### X-ray micro-computed tomography

X-ray $\mu$CT is a non-destructive imaging technique that allows for the characterization of the internal three-dimensional micro-structure of heterogeneous materials [11, 48]. In computed tomography, an X-ray source emits a beam of X-rays that passes through the specimen of interest, which is typically mounted on a rotating stage. The X-ray intensity transmitted through the specimen is projected onto an imaging detector. The degree of X-ray absorption typical of each individual phase within the sample is recorded in the detector on a regular grid of pixels to create a two-dimensional gray-scale image. During the imaging process, the specimen is slowly rotated 360 degrees around its vertical axis, such that multiple projection images are collected from different angles. The images generated of the specimens’ cross-section are commonly referred to as *slices*. As a final step, the slices are reconstructed into a regular grid of voxels to create a three-dimensional image of the meso-structure of the specimen [30].

In this work, two rectangular cuboidal oak samples were analyzed using a Phoenix Nanotom S $\mu$CT instrument. The $\mu$CT instrument was equipped with a high-resolution X-ray source generating X-rays with a voltage of 60 kV and a current of 240 $\mu$A. The two samples were cut from a larger specimen along the three anatomical (R-T, R-L, T-L) planes, and have approximate dimensions of $4 \times 6 \times 2$ mm$^3$ and $5 \times 5 \times 2$ mm$^3$. The samples were securely mounted on the rotating stage of the $\mu$CT instrument and subjected to X-rays, thus producing transmission data. The $\mu$CT scanning was performed under laboratory conditions, at a relative humidity RH $= 50 \%$ and a temperature T $= 21~^\circ$C, which define the reference state of the samples. The 2D X-ray projection images obtained from the $\mu$CT scanning process were converted to 3D volumes using Phoenix Datos|x software. Subsequently, VGStudio MAX 2.2 was employed to refine the volumetric data by adjusting the contrast and colour thresholds. The multi-channel images constructed were next converted to gray-scale images using the image processing toolbox of Matlab, by removing the hue and saturation components while preserving the luminance. The three-dimensional gray-scale images were generated using a voxel size of $1.3 \times 1.3 \times 1.3$ $\mu$m$^3$. From each of the two scanning results, a representative meso-structural region was identified that contains a single growth ring and a horizontal band of multi-seriate rays that runs along the bottom edge of the region, see Fig. 2b.

#### Construction of meso-scale domain by level set-based image segmentation

The three-dimensional gray-scale images obtained by $\mu$CT scanning are segmented to distinguish the solid phase (cell wall material) in the oak meso-structure from the void phase (lumen and vessels). The segmented output serves as input for subsequent post-processing and numerical analyses. Accordingly, in the image segmentation procedure the scanned meso-scale domain $\mathcal {Q}$ captured by the three-dimensional gray-scale image is decomposed into the two subdomains $\mathcal {Q}^+$ and $\mathcal {Q}^-$ of the respective solid and void phases. The geometries of these phases are identified by applying a segmentation method based on an evolving level set function $\phi : \mathcal {Q} \longrightarrow \mathbb {R}$ [31, 53, 69]. In order to initialize the level set function, the Euclidean distance is computed from each voxel location $\textbf{x}$ to the closest point on the phase boundary $\Gamma$ separating the two phases. This leads to the function $\text {dist}(\textbf{x}, \Gamma )$, which allows to express the level set function as a signed distance function by assigning positive and negative signs to $\text {dist}(\textbf{x}, \Gamma )$ when $\textbf{x}$ is, respectively, located in the subdomains $\mathcal {Q}^+$ and $\mathcal {Q}^-$, i.e.,

9 $$\begin{aligned} {\phi }(\textbf{x})= {\left\{ \begin{array}{ll} +\text {dist}(\textbf{x},\Gamma ) \qquad \hbox {for} \qquad \textbf{x}\in \mathcal {Q}^+ , \\ -\text {dist}(\textbf{x},\Gamma ) \qquad \hbox {for} \qquad \textbf{x}\in \mathcal {Q}^-. \end{array}\right. } \end{aligned}$$

Correspondingly, the location of the phase boundary $\Gamma$ between the subdomains $\mathcal {Q}^+$ and $\mathcal {Q}^-$ is given by

10 $$\begin{aligned} \Gamma = \{ \textbf{x} \mid \phi (\textbf{x}) = 0 \} \qquad \hbox {in} \qquad \mathcal {Q}. \end{aligned}$$

The level set function represented by equation (9) is incorporated in an energy formulation originally proposed in [13, 14] and subsequently enhanced in [75]. In addition to global image information, the enhanced energy formulation considers local image information that allows to adequately segment images with significant intensity inhomogeneity. The evolution equation of the level set function and the corresponding boundary conditions of the scanned domain are rigorously derived from the minimization of the energy formulation. The solution of the minimization problem is computed through an incremental update procedure, which converges when the desired phase boundary is reached such that everywhere in the domain $\mathcal {Q}$ the level set function becomes stationary within a predefined tolerance. The image segmentation result is finalized by locally merging accidentally disconnected subdomains of the same phase, and by assigning the void phase to small subdomains erroneously referring to a (partly) disconnected solid phase embedded within a larger void phase. For more background information on the level set-based image segmentation method, the reader is referred to [42].

**Fig. 3:**
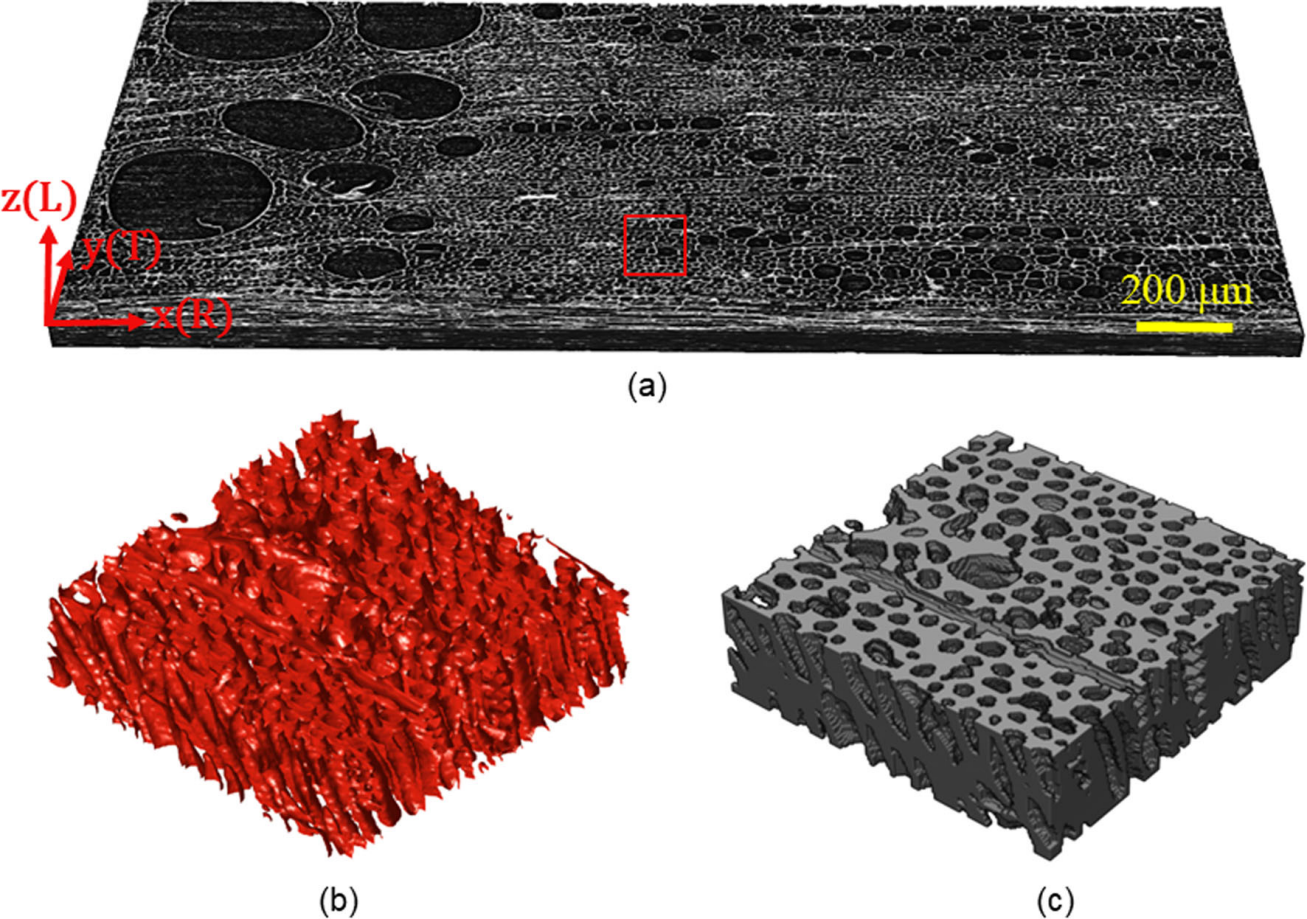
Meso-scale model: Image segmentation process for constructing the meso-scale domain *Q*. **a** Gray-scale image of an oak growth ring and a (horizontal) band of multi-seriate rays, obtained from $\mu$CT scanning. The red box denotes the part of the meso-structure that is enlarged in Fig. 3b, c. **b** Detailed view of phase boundaries $\Gamma$ between cell wall material and voids (after convergence of the image segmentation process). **c** Detail of a 3D binary image of the segmented cell wall material and voids, with the gray voxels denoting the cell wall material

Figure 3 summarizes the image segmentation process for a representative gray-scale image of an oak growth ring obtained by X-ray $\mu$CT scanning. The gray-scale image is shown in Fig. 3a, where the *x*-, *y*- and *z*-axes of the Cartesian coordinate system are, respectively, aligned with the radial (R), the tangential (T) and the longitudinal (L) material directions of the oak wood. Here, the small red box denotes the part of the oak meso-structure that is depicted enlarged in Fig. 3b, c, after the image segmentation has been performed. Figure 3b shows the phase boundaries $\Gamma$ between the cell wall material and the voids, in agreement with the zero value of the converged level set function, $\phi (\textbf{x}) = 0$, see equation (10). Figure 3c illustrates the two segmented phases by means of a 3D binary image, with the gray voxels representing the cell wall material.

The two-phase meso-structure depicted in the binary image serves as input for constructing the periodic meso-scale domain *Q* used in the asymptotic homogenization procedure, see Fig. 2b. To ensure periodicity along the *z*-direction, the segmented volume is first symmetrically mirrored across the R-T plane. Subsequently, the original and mirrored volumes at the R-T plane are merged into a single domain. For ensuring periodicity in the *x*- and *y*-directions, thin (cell wall) material layers with a thickness of two voxels are added to the sample boundaries parallel to the R-L and T-L planes. As a final step, the voxels in the 3D binary image are mapped to the eight-node hexahedral elements constructing the underlying finite element mesh. The discretization strategy is performed within a level set-based XFEM framework, which will be discussed in Sect. 4.

Table 1 summarizes the geometrical features of two meso-structural domains *Q* considered in the numerical simulations, which represent different oak growth rings and are referred to as samples A and B. The width *W*, height *H*, and thickness $\mathcal {T}$ reflect the dimensions of the unit cell *Q* along the R-, T-, and L-directions, respectively. Further, $W_e$ represents the width of the earlywood (EW) region, from which the width of the latewood region straightforwardly follows as ($W-W_e$). An illustrative example of the widths $W_e$ and ($W-W_e$) of the earlywood and latewood regions within a growth ring is given in Fig. 1a. Note from Table 1 that the aspect ratio *W*/*H* of the tested oak growth ring samples is 1.8 to 2.0; this range of values lies considerably above the aspect ratios of the oak growth ring samples analyzed in [43], which varied between 0.5 and 0.9. This difference provides a good impression of the degree of variation of the aspect ratios of growth rings in oak wood. The porosities of the samples are $\phi _A = 0.454$ and $\phi _B = 0.481$. Assuming a cell wall density in the dry state of $\rho _{cw} = 1440$ kg/m$^3$ [35], the densities $\rho = (1-\phi )\rho _{cw}$ of the two meso-scale unit cells become $\rho _\textrm{A} = 786$ kg/m$^3$ and $\rho _\textrm{B} = 748$ kg/m$^3$. These densities fall within the range of 461 kg/m$^3$ to 874 kg/m$^3$ reported in the literature for pedunculate oak [2]. In the segmented meso-structure, a distinction can be made between axial cells and ray cells, as axial cells are mainly oriented along the longitudinal material direction of oak wood and include libriform fibers, fiber tracheids and parenchyma, while ray cells, or rays, are oriented along the radial material direction. Accordingly, the solid volume fraction of ray cells is determined as $v_\textrm{ray} = V_\textrm{ray}/(V_\textrm{ax}+V_\textrm{ray})$, where $V_\textrm{ax}$ and $V_\textrm{ray}$ represent the measured volumes of axial cells and ray cells, respectively. Finally, in the earlywood (EW) region, the void volume related to vessels (oriented in the longitudinal material direction) is measured and used to compute the vessel volume fraction $v_{\textrm{vess,EW}}$ with respect to the total volume of the meso-scale unit cell.

**Table 1:** Meso-scale model: characteristics of two different meso-scale unit cells *Q*, referred to as samples A and B

Sample	Porosity	Density	Width	Height	Thickness	Ray volume fraction	Width of EW	Vessel volume fraction in EW
	$\phi$	$\rho$	*W*	*H*	$\mathcal {T}$	$v_{\textrm{ray}}$	$W_e$	$v_{\textrm{vess,EW}}$
	[–]	[kg/m$^3$]	[$\upmu$m]	[$\upmu$m]	[$\upmu$m]	[-]	[$\upmu$m]	[–]
A	0.454	786	2600	1300	65	0.21	756	0.14
B	0.481	748	2600	1469	65	0.19	1021	0.16

The unit cells contain a single growth ring and a band of multi-seriate rays, see also Fig. 2b, and are constructed from $\mu$CT images of oak meso-structures. The abbreviation “EW” refers to “earlywood”

### Constitutive behaviour

#### Meso-scale moisture-dependent hygro-elastic properties of cell walls

The cell walls defining the mesoscopic structure of oak wood have a layered structure, consisting of a primary wall and a secondary wall, with the secondary wall composed of the so-called S1, S2, and S3 layers. Similar to previous modelling studies [40, 43, 50, 62], in the present work, the cell walls are considered homogeneous orthotropic solids. The assumption of a homogeneous cell wall is reasonable, as the stiffness mismatch between the individual layers in the primary and secondary walls is moderate to small [43] so that the mechanical response of the cell wall is accurately represented by its effective stiffness properties.

**Fig. 4:**
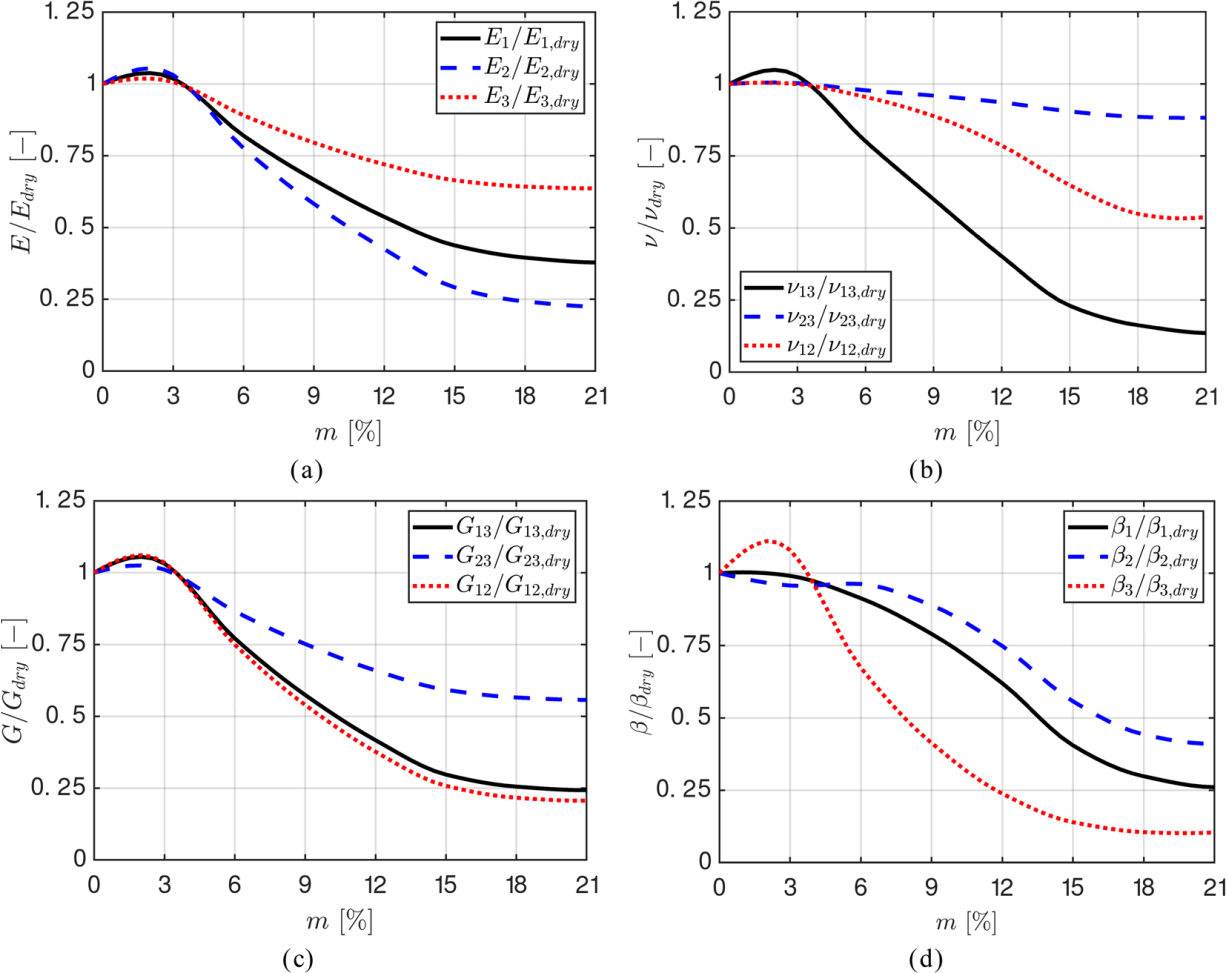
Meso-scale model: Hygro-elastic properties of cell walls of axial cells (normalized with respect to their value in the dry state) as a function of the moisture content. **a** Young’s moduli. **b** Poisson’s ratios. **c** Shear moduli. **d** Hygro-expansion coefficients

Note that cell walls may have different mechanical and physical characteristics, depending on whether they belong to libriform fibers, fiber tracheids, parenchyma, or rays. In agreement with the image segmentation procedure discussed in Sect. 3.1.2, the *axial cells*, which refer to libriform fibers, fiber tracheids, and parenchyma, are assumed to have the same cell wall properties. In contrast, *ray cells* oriented in the radial direction have different cell wall properties. For a cell wall *cw*, with $cw \in \{\textrm{axial}, \textrm{ray}$}, the orthotropic fourth-order compliance tensor $^4\textbf{D}^{cw}$ and the second-order hygro-expansion tensor $\varvec{\beta }^{cw}$ are given by:

11 $$\begin{aligned}  ^4\textbf{D}^{cw}= &   \dfrac{1}{E_{1}}\textbf{e}_1\otimes \textbf{e}_1\otimes \textbf{e}_1\otimes \textbf{e}_1 - \dfrac{\nu _{12}}{E_{1}} \textbf{e}_1\otimes \textbf{e}_1\otimes \textbf{e}_2\otimes \textbf{e}_2 \nonumber \\  &   - \dfrac{\nu _{13}}{E_{1}} \textbf{e}_1\otimes \textbf{e}_1\otimes \textbf{e}_3\otimes \textbf{e}_3 - \dfrac{\nu _{12}}{E_{1}} \textbf{e}_2\otimes \textbf{e}_2\otimes \textbf{e}_1\otimes \textbf{e}_1\nonumber \\  &   + \dfrac{1}{E_{2}}\textbf{e}_2\otimes \textbf{e}_2\otimes \textbf{e}_2\otimes \textbf{e}_2- \dfrac{\nu _{23}}{E_{2}} \textbf{e}_2\otimes \textbf{e}_2\otimes \textbf{e}_3\otimes \textbf{e}_3 \nonumber \\  &   - \dfrac{\nu _{13}}{E_{1}} \textbf{e}_3\otimes \textbf{e}_3\otimes \textbf{e}_1\otimes \textbf{e}_1 - \dfrac{\nu _{23}}{E_{2}} \textbf{e}_3\otimes \textbf{e}_3\otimes \textbf{e}_2\otimes \textbf{e}_2\nonumber \\  &   + \dfrac{1}{E_{3}}\textbf{e}_3\otimes \textbf{e}_3\otimes \textbf{e}_3\otimes \textbf{e}_3 + \dfrac{1}{G_{12}}\textbf{e}_1\otimes \textbf{e}_2\otimes \textbf{e}_1\otimes \textbf{e}_2 \nonumber \\  &   + \dfrac{1}{G_{23}}\textbf{e}_2\otimes \textbf{e}_3\otimes \textbf{e}_2\otimes \textbf{e}_3 + \dfrac{1}{G_{13}}\textbf{e}_1\otimes \textbf{e}_3\otimes \textbf{e}_1\otimes \textbf{e}_3, \nonumber \\ \varvec{\beta }^{cw}= &   \beta _1 \textbf{e}_1\otimes \textbf{e}_1 + \beta _2\textbf{e}_2\otimes \textbf{e}_2 + \beta _3\textbf{e}_3\otimes \textbf{e}_3 ~, \end{aligned}$$

where the compliance tensor $^4\textbf{D}^{cw}$ is related to the stiffness tensor $^4\textbf{C}^{cw}$ via the relation $^4\textbf{D}^{cw} = \left( ^4\textbf{C}^{cw}\right) ^{-1}$. Note that Eq.(11) incorporates the orthotropic symmetry conditions for the off-diagonal terms, i.e., ${{\nu }_{12}}/{{E}_{1}} = {{\nu }_{21}}/{{E}_{2}}$, ${{\nu }_{13}}/{{E}_{1}} = {{\nu }_{31}}/{{E}_{3}}$ and ${{\nu }_{23}}/{{E}_{2}} = {{\nu }_{32}}/{{E}_{3}}$. Hence, the orthotropic stiffness tensor $^4\textbf{C}^{cw}$ is composed of nine independent elastic parameters, see e.g., [60], which, as shown by equation (11), are here specified via the elastic moduli ($E_{1}, E_2, E_3$), Poisson’s ratios ($\nu _{12}, \nu _{23}, \nu _{13}$) and shear moduli ($G_{12}, G_{23}, G_{13}$). Additionally, the hygro-expansion tensor $\varvec{\beta }^{cw}$ is characterized by the three hygro-expansion coefficients ($\beta _{1}, \beta _2, \beta _3$).^1^ Although not explicitly indicated in equation (11), as mentioned in Sect. 2, these hygro-elastic parameters depend on the moisture content. The moisture content is considered uniform at the meso-scale level and is controlled by the ambient relative humidity through the moisture sorption isotherms. Figure 4 illustrates the cell wall hygro-elastic properties (normalized with respect to their value in the dry state) as a function of the moisture content *m*. These properties were computed with the multi-scale model for oak wood presented in [43], which incorporates nano-scale structural characteristics, and considers cell walls with a microfibril angle of the S2 layers of 20$^\circ$. Since the material properties of the axial and ray cell walls exhibit similar moisture-dependent trends [43], only the results for the axial cell walls are depicted for brevity. At the reference moisture content $m=12\%$, the hygro-elastic cell wall properties of axial and ray cell walls are summarized in Table 2. The hygro-elastic parameters of the cell walls can be determined at an arbitrary value of the moisture content *m*, by scaling the reference properties in Table 2 using the ratio of the corresponding value in Fig. 4 at the moisture content *m* to its value at $m = 12\%$.

**Table 2:** Meso-scale model: effective hygro-elastic parameters of cell walls, corresponding to a reference moisture content ${m} = 12\%$

	Young’s modulus [GPa]			Poisson’s ratio [–]			Shear modulus [GPa]		
Cell type	${E}_{1}$	${E}_{2}$	${E}_{3}$	${\nu }_{23}$	${\nu }_{13}$	${\nu }_{12}$	${G}_{23}$	${G}_{13}$	${G}_{12}$
Axial cells	5.8	3.7	18.3	0.02	0.19	0.24	1.2	4.4	1.0
Ray cells	4.9	3.5	13.0	0.01	0.30	0.21	1.1	5.6	1.0

	Hygro-expansion coefficient [%/%]								
Cell type	${\beta }_{1}$	${\beta }_{2}$	${\beta }_{3}$						
Axial cells	0.16	0.40	0.01						
Ray cells	0.17	0.43	0.00						

These properties are defined with respect to a local (1, 2, 3) Cartesian coordinate system directed along the principal material directions of the cell walls, and are computed from the structural characteristics at the nano-scale using the multi-scale model for oak wood presented in [43], where the microfibril angle of the S2 layers of the axial cells and ray cells is taken to be the same and equals 20$^{\circ }$

#### Identification of the local material orientation in cell walls

The meso-scale properties listed in Table 2 refer to the local principal (1, 2, 3) material directions of a cell wall. These properties need to be converted to the global reference system (*x*, *y*, *z*) at the meso-scale (shown in Fig. 2b) to perform the asymptotic homogenization procedure. For this purpose, it is assumed that each hexahedral finite element of the discretized meso-scale domain is characterized by a single value for the material orientation, as computed at the geometrical center of the element. For the axial cells, the (vertical) $3-$axis of the cell wall coincides with the longitudinal material direction corresponding with the global $z-$axis. Accordingly, only the local $1-$ and $2-$ directions need to be identified and expressed with respect to the $x-$ and $y-$ directions of the global reference system at the meso-scale. Recall that the level set function $\phi (x,y,z)$ defined in equation (9) represents the Euclidean distance of a material point (located at the center of a voxel) to the closest point on a (cell wall-void) phase boundary. Hence, the spatial gradient of the level set function $\phi$ can be used for approximating the local material orientation in the cell walls in the following way. For each planar $x-y$ section of the meso-scale domain, at a given vertical coordinate $z= \hat{z}$ the components of the spatial gradients $\phi (x,y,\hat{z})_{,x}$ and $\phi (x,y,\hat{z})_{,y}$ are evaluated using a central difference scheme. Subsequently, the material orientation at a location in the $x-y$ plane is described by the angle $\alpha (x,y,\hat{z})$ between the global $x-$axis and the local $1-$direction as

12 $$\begin{aligned} \alpha (x,y,\hat{z}) = \arctan \left( \frac{-\phi (x,y,\hat{z})_{,x}}{\phi (x,y,\hat{z})_{,y}}\right) \, . \end{aligned}$$

Using the angle $\alpha (x,y,\hat{z})$, the meso-scale material properties listed in Table 2 can be expressed in terms of the $x-$ and $y-$ directions of the global meso-scale reference system by applying a standard coordinate transformation. For the ray cells, a similar procedure is followed, where the (vertical) $3-$axis of the ray cell wall coincides with the radial material direction that corresponds with the global $x-$axis.

**Fig. 5:**
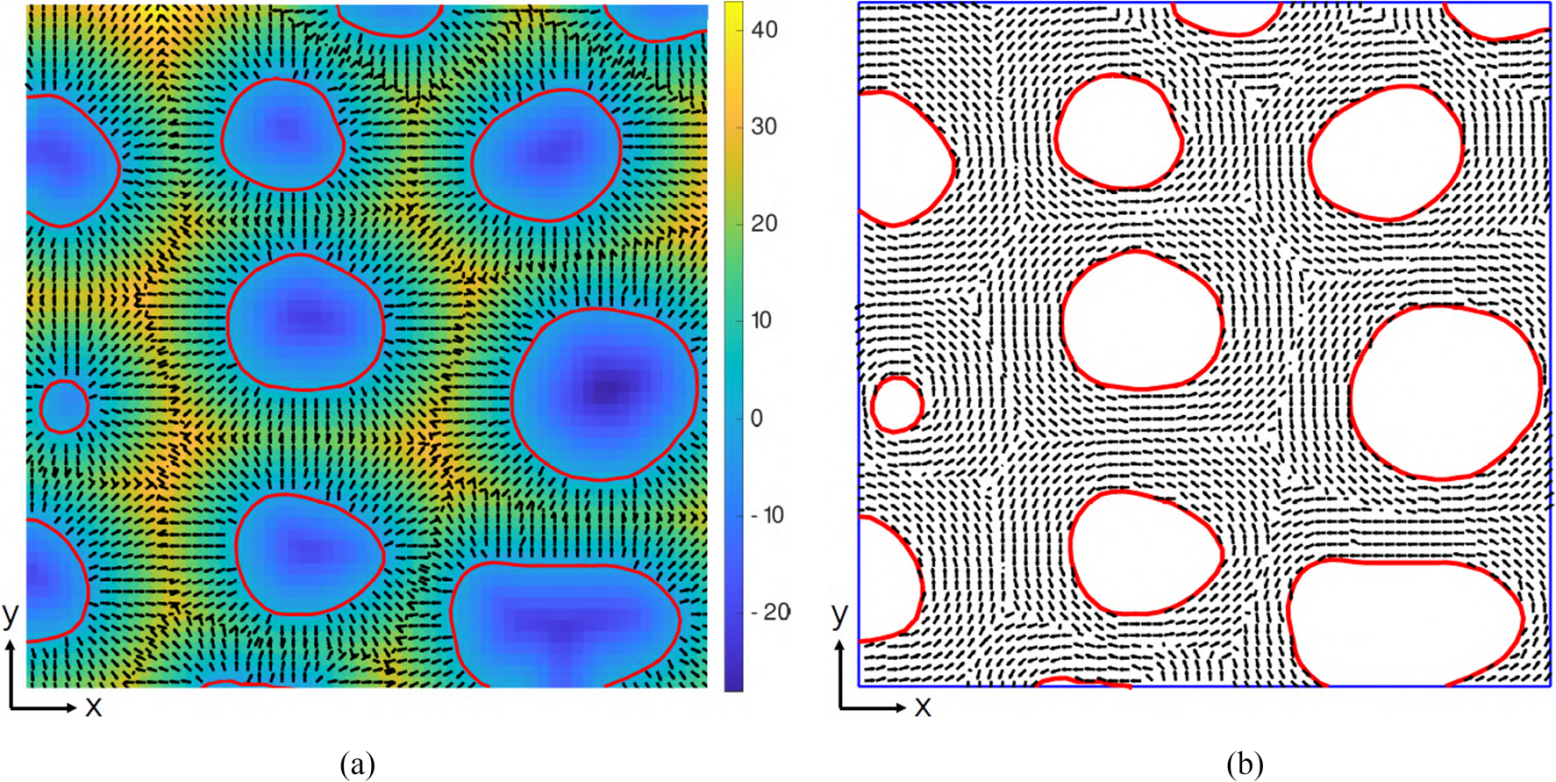
Meso-scale model: Identification of the local material orientation of axial cells, illustrated on a selected region of an $x-y$ cross-section of an oak wood sample with a vertical coordinate $z=\hat{z}$. **a** Contour plot of the level set function $\phi (x,y,\hat{z})$, with the red solid lines representing the phase boundaries between the cell walls and the voids. The small black arrows denote the vector field corresponding to the spatial gradient of $\phi (x,y,\hat{z})$. **b** Cell wall material orientation, with the black solid lines denoting the directions of the local $1-$axes in the $x-y$ plane

Figure 5 illustrates the two steps performed for the identification of the local material orientations in the axial cells. Figure 5a shows a selected region of an $x-y$ cross-section at a vertical coordinate $z=\hat{z}$. The contour plot illustrates the value of the level set function $\phi (x,y,\hat{z})$, and the red solid lines represent the phase boundaries between the cell walls and the voids. The small black arrows denote the vector field corresponding to the spatial gradient of $\phi (x,y,\hat{z})$. Figure 5b shows the cell wall material orientation, with the black solid lines denoting the directions of the local $1-$axes in the $x-y$ plane.

## Numerical solution procedure

### Level set-based XFEM method

The geometrical complexity of the three-dimensional meso-structures of oak wood considered in this work makes it computationally inefficient to discretize the domain with a geometry-conforming mesh using the classical finite element method, due to the large number of elements required [19]. Hence, the extended finite element method (XFEM) is employed instead, which is a numerical discretization method that accurately and efficiently describes material discontinuities, singularities and phase boundaries, by allowing these to run arbitrarily through the underlying (non-conforming) finite element mesh [24]. In the XFEM formulation used in the present work, the geometrical discontinuities between cell walls and voids are captured by enriching the classical shape functions with a Heaviside function defined in terms of the level set function $\phi (\textbf{x})$ [15, 41]. As pointed out in Sect. 3.1.2, the level set function already defines the locations of the phase boundaries in the image segmentation method, so that it can be straightforwardly applied as the governing variable in the Heaviside enrichment function to represent the locations of the phase boundaries in the XFEM approach. Accordingly, for a meso-scale domain *Q* discretized by a non-conforming mesh composed of $n_e$ elements, for a finite element *e*, the interpolations of the meso-scale influence functions $^3\textbf{N}_{1}$ and $\textbf{b}_{1}$, which define the meso-scale displacement field $\textbf{u}_1(\textbf{x})$ via equation (5), are expressed by [71]

13 $$\begin{aligned} \begin{array}{rcl} ^3\textbf{N}_{1}^e(\textbf{x}) & = &  \displaystyle { \sum _{i =1 }^{n_{n}} H \! \left( \phi (\textbf{x}) \right) {\zeta }^i(\textbf{x}) \, ^3\textbf{N}_{1}^i ~,} \\ \textbf{b}_{1}^e(\textbf{x}) & = &  \displaystyle { \sum _{i =1 }^{n_{n}} H \! \left( \phi (\textbf{x}) \right) {\zeta }^i(\textbf{x}) \, \textbf{b}_{1}^i ~,} \end{array} \end{aligned}$$

where $n_{n}$ represents the number of nodes in element *e*, $\zeta ^i$ is the finite element shape function related to node *i*, and $^3\textbf{N}_{1}^i$ and $\textbf{b}_{1}^i$ are the nodal values of the influence functions. The shape functions ${\zeta }^i$, with $i = 1,.., n_n$, are represented by the standard shape functions of eight-node hexahedral elements, so that $n_n = 8$. Further, with the definition of the level set function $\phi (\textbf{x})$ in equation (9), the Heaviside function $H \! \left( \phi (\textbf{x}) \right)$ is given by

14 $$\begin{aligned} H \! \left( \phi (\textbf{x}) \right) = {\left\{ \begin{array}{ll} 1 &  \text {if } \phi (\textbf{x}) \ge 0 ~, \\ 0 &  \text {if } \phi (\textbf{x}) < 0 ~. \end{array}\right. } \end{aligned}$$

In the meso-scale domain *Q*, the influence functions $^3\textbf{N}_{1}$ and $\textbf{b}_1$ are computed numerically by solving the cell problems given by equations (4)$_{1,2}$. This is done by using the weak form of these equations, i.e.,15with $Q_e$ referring to finite element *e* with volume $|Q_e|$, and $^3\varvec{\mathfrak {N}}(\textbf{x})$ and $\varvec{\mathfrak {b}}(\textbf{x})$ the third-order tensor and vector test functions, respectively. Equations (15)$_{1,2}$ are further developed using a compact “column matrix” notation. Accordingly, the nodal degrees of freedom $\textbf{N}_{1}^i, \textbf{b}_{1}^i$ and the shape functions ${\zeta }^i(\textbf{x})$ related to the nodes are stored in column matrices of the eight-node hexahedral elements (where a “column matrix" is indicated by a tilde below the corresponding matrix symbol):16which allows to rewrite equations (13)$_{1,2}$ as:17Similarly, the gradients $\varvec{\nabla }{~^3\textbf{N}}^{e}_{1}(\textbf{x})$ and $\varvec{\nabla }\textbf{b}^{e}_{1}(\textbf{x})$ that appear in equations (15)$_{1,2}$ can be expressed as18where the column of vectors  is defined as19According to the standard Galerkin approach, the shape functions  can be used to compute the test functions $^3\varvec{\mathfrak {N}}(\textbf{x})$ and $\varvec{\mathfrak {b}}(\textbf{x})$ in equations (15)$_{1,2}$ as20and their gradients as21where the columns  and  contain the corresponding nodal values. Inserting equations (17) to (21) into equations (15)$_{1,2}$, and taking into account that the resulting expressions must be satisfied for arbitrary test functions, leads to the following system of linear equations:22in which the columns  and  contain the unknown nodal values of the meso-scale fluctuation functions. Additionally, the second-order stiffness tensor $\underline{\textbf{K}}$ (which, from hereon, will be referred to as the “stiffness matrix”) and the third-order “loading” tensor  and the “loading” vector  are given by23The meso-scale fluctuation functions  and  appearing in equation (22) satisfy periodic boundary conditions, in accordance with24where superscripts *l*, *r*, *t*, *d*, *f*, *b* refer to the boundary surfaces of the unit cell, i.e., left, right, top, down, front and back, respectively.

**Fig. 6:**
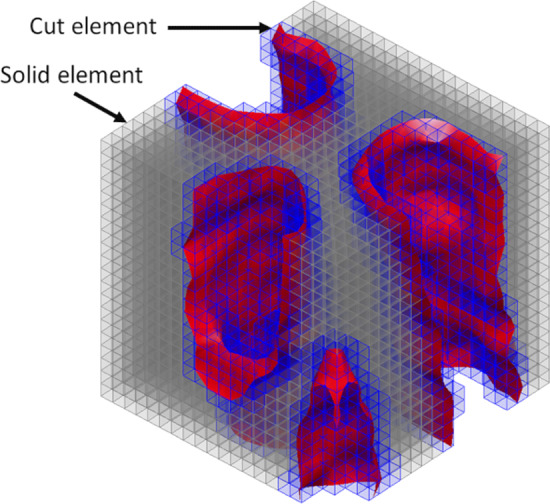
Numerical solution procedure: A small part of the meso-scale domain *Q* after the discretization by eight-node hexahedral elements. The phase boundaries corresponding to a zero value of the level set function, $\phi (\textbf{x})=0$, are depicted in red. The elements for which $\phi (\textbf{x})>0$ are *solid* (cell wall) elements and are shown in gray. The elements for which $\phi (\textbf{x})<0$ are *void* elements and are not depicted for clarity. The elements that intersect with the phase boundaries are *cut* elements and are shown in blue

Figure 6 shows a small part of the meso-scale domain *Q* after applying the discretization by eight-node hexahedral elements. The phase boundaries corresponding to a zero value of the level set function, $\phi (\textbf{x})=0$, are depicted in red. The elements for which $\phi (\textbf{x})>0$ are *solid* (cell wall) elements and are shown in gray. For these elements, the Heaviside function $H\!\left( \phi (\textbf{x})\right)$ in equations (23)$_{1,2,3}$ is equal to unity. Consequently, the full element contribution is accounted for in the computation of the overall stiffness matrix $\underline{\textbf{K}}$, the loading tensor  and the loading vector . Accordingly, the integration over the element volumes $Q_e$ is performed with a standard Gaussian quadrature. The elements for which $\phi (\textbf{x})< 0$ are *void* elements and are not depicted in Fig. 6 for clarity. For these elements, the Heaviside function $H\!\left( \phi (\textbf{x})\right)$ in equations (23)$_{1,2,3}$ equals zero so that they do not contribute to the overall stiffness matrix, the loading tensor and the loading vector. Finally, the elements that intersect with the phase boundaries are *cut* elements, which are shown in blue. These elements contribute to the overall stiffness tensor, loading tensor and loading vector and require a specific integration procedure, as discussed in Sect. 4.2 below.

### Efficient numerical integration of *cut* elements

For determining the stiffness matrix $\underline{\textbf{K}}$ and the loading terms  and  defined by equations (23)$_{1,2,3}$, the integrals in these expressions are computed by using numerical quadrature. Accordingly, for an eight-node hexahedral isoparametric element, the element stiffness matrix $\underline{\textbf{K}}^e$ and the loading terms  and  are calculated as25where $\varvec{\xi }_i$, with $i = 1,...n_{i}$, are the locations of the integration points in the isoparametric elements and $w_i$ are the corresponding weight factors. The term $\textrm{det} \, \textbf{J}$ reflects the determinant of the Jacobian tensor, which appears due to the coordinate transformation from the physical space to the isoparametric space. As already mentioned, for the *solid* (cell wall) elements the integration points and the weight factors are selected in correspondence with a standard Gaussian quadrature, where an eight-point ($n_{i}=8$) integration scheme is used for the hexahedral elements.

**Fig. 7:**
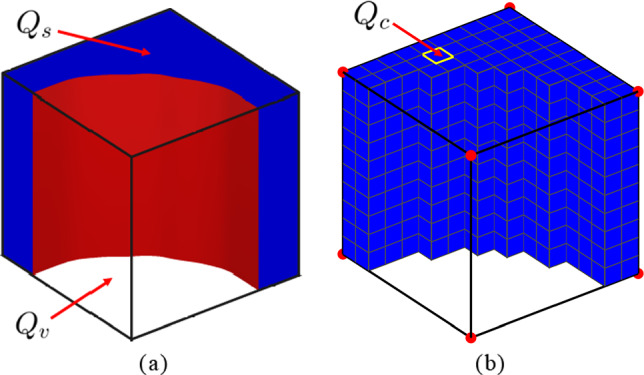
Numerical solution procedure: Refined discretization of the solid (cell wall) domain within a *cut* element, in order to determine its quadrature weights $w_i$ by moment fitting. **a** Typical *cut* element composed of a solid domain $Q_s$ (depicted in blue) and a void domain $Q_v$ (transparent), separated by a phase boundary (depicted in red) that corresponds to a zero value of the level set function, $\phi (\textbf{x})=0$. **b** Discretization of a *cut* element through a refined structured mesh of $n_c=10^3$ sub-cells of volume $Q_c$. Only the sub-cells in the solid domain $Q_s$ (depicted in blue) contribute to the determination of the quadrature weights $w_i$ of the *cut* element, in correspondence with a unity value $H^c=1$ of the Heaviside function in equation (31)

For the *cut* elements, which are intersected by the boundaries of the cell walls, the so-called *moment fitting* integration method is employed to accurately compute the weight factors $w_i$ in expressions (25)$_{1,2,3}$. This is done by following the approach presented in [33, 52]. First, the locations $\varvec{\xi }_i$ of the $n_{i}$ integration points are selected in accordance with a Gaussian quadrature. At the element level, the evaluations of the basis functions, $g_j(\varvec{\xi _i})$, with $j = 1,..., n_{g}$, where $n_g$ represents the total number of basis functions, together with the corresponding quadrature weights $w_i$ satisfy the moment equation defining the usual quadrature rule:

26 $$\begin{aligned} \sum _{i=1}^{n_{i}} g_j(\varvec{\xi }_i) \, w_i= &   \int _{Q_s} g_j(\varvec{\xi }) \, \textrm{d} Q_s \nonumber \\= &   \int _{Q_e} H \! \left( \phi (\varvec{\xi }) \right) g_j(\varvec{\xi }) \, \textrm{d} Q_e \quad \forall ~j = 1,..., n_{g} ~, \nonumber \\ \end{aligned}$$

where the total element domain $Q_e = Q_s \cup Q_v$ is composed of a *solid* (cell wall) part $Q_s$ and a *void* part $Q_v$, see Fig. 7a. Following [33], in equation ((26) the total number of integration points is set equal to the total number of basis functions, $n_{i}=n_{g}$. Hence, the expressions given by equation (26) reduce to a system of linear equations, where the components $g_j(\varvec{\xi }_i)$ can be conveniently captured in a square matrix. Further, the relation between the number of integration points $n_{i}$, the number of basis functions $n_{g}$, the quadrature order $p_q$, and the spatial dimension *d* of the problem under consideration reads [33]

27 $$\begin{aligned} n_{i} = n_{g} = (p_q+1)^d ~. \end{aligned}$$

The basis functions $g_j(\varvec{\xi })$ are here defined by Lagrange polynomials $\ell _j(\varvec{\xi })$, i.e.,

28 $$\begin{aligned}  &   g_j(\varvec{\xi }) = \ell _j(\varvec{\xi }) \, \nonumber \\  &   \text { with } \quad \ell _j(\varvec{\xi })= \prod _{\begin{array}{c} k=1 \\ k\ne j \end{array}}^{(p_q+1)^d} \frac{\xi _{r} - \xi _{r,k}^{GL}}{\xi _{r,j}^{GL} - \xi _{r,k}^{GL}} \prod _{\begin{array}{c} k=1 \\ k \ne j \end{array}}^{(p_q+1)^d} \frac{\xi _{s} - \xi _{s,k}^{GL}}{\xi _{s,j}^{GL} - \xi _{s,k}^{GL}}\nonumber \\    &   \quad \times \prod _{\begin{array}{c} k=1 \\ k \ne j \end{array}}^{(p_q+1)^d} \frac{\xi _{t} - \xi _{t,k}^{GL}}{\xi _{t,j}^{GL} - \xi _{t,k}^{GL}} ~~ \quad ~\forall ~j = 1,..., n_{g} ~, \end{aligned}$$

where $\prod$ is the product operator, $\xi _{r}, \xi _{s}$ and $\xi _{t}$ are the components of $\varvec{\xi }$ in the local Cartesian basis $\{ \textbf{e}_r,\textbf{e}_s, \textbf{e}_t \}$ of the isoparametric element, and $\varvec{\xi }_k^{GL} = ({\xi }_{r,k}^{GL}, {\xi }_{s,k}^{GL}, {\xi }_{t,k}^{GL})$ are the locations of the Gauss integration points. Due to the orthogonality property of Lagrange polynomials, it follows that

29 $$\begin{aligned} \ell _j(\varvec{\xi }^{GL}_i) = \delta _{ji} ~. \end{aligned}$$

Combining equations (29) and (28) with equation (26) leads to an *explicit* expression for the quadrature weights $w_i$, i.e.,

30 $$\begin{aligned} w_i = \int _{Q_e} H \! \left( \phi ( \varvec{\xi }) \right) \ell _i(\varvec{\xi }) \, \textrm{d} Q_e \qquad \forall ~i = 1, ..., n_{i} ~. \end{aligned}$$

As shown by expression (30), the weight factors $w_i$ in a *cut* element depend on the actual geometry of the phase boundary, defined through the Heaviside function $H \! \left( \phi (\varvec{\xi })\right)$. In this work, the integral expression (30) is accurately computed by subdividing the *cut* elements in a large number $n_c$ of equal cubic sub-cells of volume $Q_{c}$, see Fig. 7b. For each sub-cell *c*, a unique value $H^c$ of the Heaviside function is assigned (that is either equal to zero or one), which is determined by the value of the level set function evaluated at the center of the sub-cell. The quadrature weights $w_i$ following from moment fitting then become

31 $$\begin{aligned}  &   w_i = \sum _{c =1}^{n_c} \int _{Q_{c}} H \! \left( \phi (\varvec{\chi }) \right) \ell _i(\varvec{\chi }) \, \textrm{d} Q_{c}\nonumber \\  &   \qquad = \sum _{c =1}^{n_c} H^c \sum _{h=1}^{8} v_h \, \ell _i(\varvec{\chi }_h)\quad \forall ~ i = 1,..., n_{i} ~, \end{aligned}$$

where $\varvec{\chi }_h$ (with $h = 1,... \, 8$) are the locations of the integration points in the isoparametric hexahedral element defining a sub-cell and $v_h$ are the weight factors, which are selected in accordance with a standard Gaussian quadrature. Note that the term $\sum _{h=1}^{8} v_h \ell _i(\varvec{\chi }_h)$ results in a scalar value that is the same for all sub-cells and thus only needs to be calculated once, making the procedure computationally efficient. After the weights $w_i$ are calculated, they can be used to compute the element stiffness matrix $\underline{\textbf{K}}^e$ and the loading terms  and  in expressions (25)$_{1,2,3}$.

The weight factors $w_i$ are computed assuming a quadrature order of $p_q = 2$ for the Lagrange polynomials in equation (28). Considering that the dimension of the problem is $d = 3$, it follows from equation (27) that the number of integration points and basis functions is $n_i = n_g = 27$. Further, the number of sub-cells within a *cut* element is taken as $n_c = 10^3$. Additional simulations not presented here have demonstrated that with these parameter values the computational time of the present approach is substantially less compared to that of a “local mesh refinement approach” where the stiffness and loading contributions of the solid sub-cells in each *cut* element (as visualized in Fig. 7b) are straightforwardly accounted for in the assemblage of the overall stiffness matrix $\underline{\textbf{K}}$ and the loading terms  and  of the modeled structure.

### Iterative solution procedure with preconditioning

From the element stiffness matrix $\underline{\textbf{K}}^e$ and loading terms  and  given by equations (25)$_{1,2,3}$, the assemblages of the overall stiffness matrix and loading terms follow as32where  denotes the element assemblage procedure. Using the above expressions, the most obvious solution strategy would be to solve the system of linear equations (22)$_{1,2}$ in a direct fashion by (stiffness) matrix inversion to obtain the nodal values of the meso-scale fluctuation functions  and . However, the XFEM discretization may result in possible ill-conditioning of the stiffness matrix when the discontinuities (i.e., phase boundaries) are located at a small distance from the nodes of continuum elements [39]. Considering the complex three-dimensional geometry of the oak wood meso-scale domains analyzed in this work, such occurrences can not be unconditionally excluded. To avoid numerical problems associated with an ill-conditioning of the stiffness matrix, the discretized set of equations (22)$_{1,2}$ is solved in an *iterative* fashion using a preconditioned conjugate gradient method. For this purpose, a preconditioner $\underline{\textbf{S}}$ is constructed that represents a sparse approximation of the inverse of the stiffness matrix $\underline{\textbf{K}}^{-1}$, as obtained by the inversion and subsequent summation of *sub-matrices* of the overall stiffness matrix ${\underline{\textbf{K}}}$ [34]. In this work, the element stiffness matrix ${\underline{\textbf{K}}}^e$ given by equation (25)$_1$ is selected as the sub-matrix, leading to the following form of the preconditioner $\underline{\textbf{S}}$:33Since the second-order tensor $\underline{\textbf{K}}^{e}$ defining the element stiffness matrix is symmetric, the spectral decomposition of $\underline{\textbf{K}}^{e}$ follows the usual expression

34 $$\begin{aligned} \underline{\textbf{K}}^{e} = \sum _{i=1}^{m_s}\lambda _i\varvec{\upsilon }_i\otimes \varvec{\upsilon }_i \qquad \textrm{and}\qquad (\underline{\textbf{K}}^{e})^{-1} =\sum _{i=1}^{m_s} \lambda _i^{-1} \, \varvec{\upsilon }_i\otimes \varvec{\upsilon }_i~, \end{aligned}$$

where $m_s$ denotes the degrees of freedom of the element stiffness matrix $\underline{\textbf{K}}^{e}$, and $\lambda _i$ and $\varvec{\upsilon }_i$ are the *i*-*th* eigenvalue and eigenvector, respectively. To ensure that the preconditioner $\underline{\textbf{S}}$ is positive definite, it is required that the eigenvalues $\lambda _i$ are strictly positive. In general, this may not always be the case, for example, when small eigenvalues - associated with *cut* elements with a small solid phase - that theoretically should be positive, become negative because of round-off errors caused by finite precision [34]. The requirement of strictly positive eigenvalues, however, can be rigorously satisfied by replacing the inverse $(\underline{\textbf{K}}^{e})^{-1}$ in equation (33) by a pseudo inverse $(\underline{\textbf{K}}_{+}^e)^{-1}$ of the form [34]

35 $$\begin{aligned} (\underline{\textbf{K}}_{+}^e)^{-1} =\sum _{i=1}^{m_s} \lambda _{i+}^{-1} \, \varvec{\upsilon }_i\otimes \varvec{\upsilon }_i~, \end{aligned}$$

with

36 $$\begin{aligned} \lambda _{i+}^{-1} = {\left\{ \begin{array}{ll} \lambda _{i}^{-1} &  \forall \, \lambda _{i}>\lambda _{\text {th}} \, ,\\ 0 &  \forall \, \lambda _{i}\le \lambda _{\text {th}} \, , \end{array}\right. } \end{aligned}$$

where the small, positive threshold value $\lambda _{\text {th}}$ is given by $\lambda _{\text {th}} =\epsilon ~ \textrm{max}_i({\lambda }_i)$, with the scalar value $\epsilon$ selected as $10^{-13}$ to ensure that the smallest eigenvalue that is inverted is sufficiently larger than the machine precision. With the use of equation (35), the stabilized preconditioner $\underline{\textbf{S}}$ given by equation (33) is applied using left preconditioning in combination with a conjugate gradient iterative solver to compute the solution of the system of linear equations (22)$_{1,2}$ in a numerically robust fashion.

### Computation of effective hygro-elastic properties

Once the nodal values of the influence functions  and  are computed, the effective hygro-mechanical properties $^{4}\bar{\textbf{C}}$ and $\bar{\varvec{\beta }}$ can be determined from the discretized form of equations (8)$_{1,2}$, which is obtained by using the (extended) finite element interpolation functions and integration strategy discussed in Sects. 4.1 to 4.2, i.e.,37where |*Q*| is the total volume of the unit cell. As described in Sect. 4.2, the quadrature weights $w_i$ are defined by standard Gauss integration for *solid* elements and by moment fitting integration for *cut* elements.

## Computational results

### Effective hygro-elastic response of oak wood

The effective hygro-elastic response of oak at the macro-scale is obtained from asymptotic homogenization of the material response at the meso-scale level, using as input the $\mu$CT scanned oak growth ring samples A and B introduced in Sect. 3.1. The meso-scale characteristics of the two samples have been discussed in Sects. 3.2 and 3.2.2 and depend on the moisture content in accordance with the trends depicted in Fig. 4. The macro-scale stiffness properties $^4\bar{\textbf{C}}$ and hygroscopic expansion coefficients $\bar{\varvec{\beta }}$ of the samples are calculated via equations (37)$_{1,2}$, respectively.

#### Elastic properties

The macroscopic elastic response of oak derived from the asymptotic homogenization procedure is (nearly) orthotropic. Accordingly, the macroscopic compliance tensor $^4\bar{\textbf{D}} =  ^4\bar{\textbf{C}}^{-1}$ can be expressed as,

38 $$\begin{aligned}  &    ^4\bar{\textbf{D}} = \dfrac{1}{\bar{E}_{R}}\textbf{e}_x\otimes \textbf{e}_x\otimes \textbf{e}_x\otimes \textbf{e}_x - \dfrac{\bar{\nu }_{RT}}{\bar{E}_{R}} \textbf{e}_x\otimes \textbf{e}_x\otimes \textbf{e}_y\otimes \textbf{e}_y \nonumber \\  &   \qquad - \dfrac{\bar{\nu }_{RL}}{\bar{E}_{R}} \textbf{e}_x\otimes \textbf{e}_x\otimes \textbf{e}_z\otimes \textbf{e}_z - \dfrac{\bar{\nu }_{RT}}{\bar{E}_{R}} \textbf{e}_y\otimes \textbf{e}_y\otimes \textbf{e}_x\otimes \textbf{e}_x\nonumber \\  &   \qquad + \dfrac{1}{\bar{E}_{T}}\textbf{e}_y\otimes \textbf{e}_y\otimes \textbf{e}_y\otimes \textbf{e}_y - \dfrac{\bar{\nu }_{TL}}{\bar{E}_{T}} \textbf{e}_y\otimes \textbf{e}_y\otimes \textbf{e}_z\otimes \textbf{e}_z \nonumber \\  &   \qquad - \dfrac{\bar{\nu }_{RL}}{\bar{E}_{R}} \textbf{e}_z\otimes \textbf{e}_z\otimes \textbf{e}_x\otimes \textbf{e}_x - \dfrac{\bar{\nu }_{TL}}{\bar{E}_{T}} \textbf{e}_z\otimes \textbf{e}_z\otimes \textbf{e}_y\otimes \textbf{e}_y\nonumber \\  &   \qquad + \dfrac{1}{\bar{E}_{L}}\textbf{e}_z\otimes \textbf{e}_z\otimes \textbf{e}_z\otimes \textbf{e}_z + \dfrac{1}{G_{RT}}\textbf{e}_x\otimes \textbf{e}_y\otimes \textbf{e}_x\otimes \textbf{e}_y \nonumber \\  &   \qquad + \dfrac{1}{G_{LT}}\textbf{e}_y \otimes \textbf{e}_z\otimes \textbf{e}_y\otimes \textbf{e}_z + \dfrac{1}{G_{LR}}\textbf{e}_x\otimes \textbf{e}_z\otimes \textbf{e}_x\otimes \textbf{e}_z ~, \end{aligned}$$

in which the orthotropic symmetry conditions for the off-diagonal terms have been accounted for, i.e., ${\bar{\nu }_{RT}}/{\bar{E}_{R}} = {\bar{\nu }_{TR}}/{\bar{E}_{T}}$, ${\bar{\nu }_{RL}}/{\bar{E}_{R}} = {\bar{\nu }_{LR}}/{\bar{E}_{L}}$ and ${\bar{\nu }_{TL}}/{\bar{E}_{T}} = {\bar{\nu }_{LT}}/{\bar{E}_{L}}$. The nine independent elastic constants appearing in equation (38) are determined by equating the components in this expression to the corresponding components of the inverse of the stiffness tensor, $^4\bar{\textbf{C}}^{-1}$, as computed from equation (37)$_1$. If necessary, from these values the Poisson’s ratios $\bar{\nu }_{TR}$, $\bar{\nu }_{LT}$ and $\bar{\nu }_{LR}$ can be computed using the orthotropic symmetry conditions mentioned above.

**Fig. 8:**
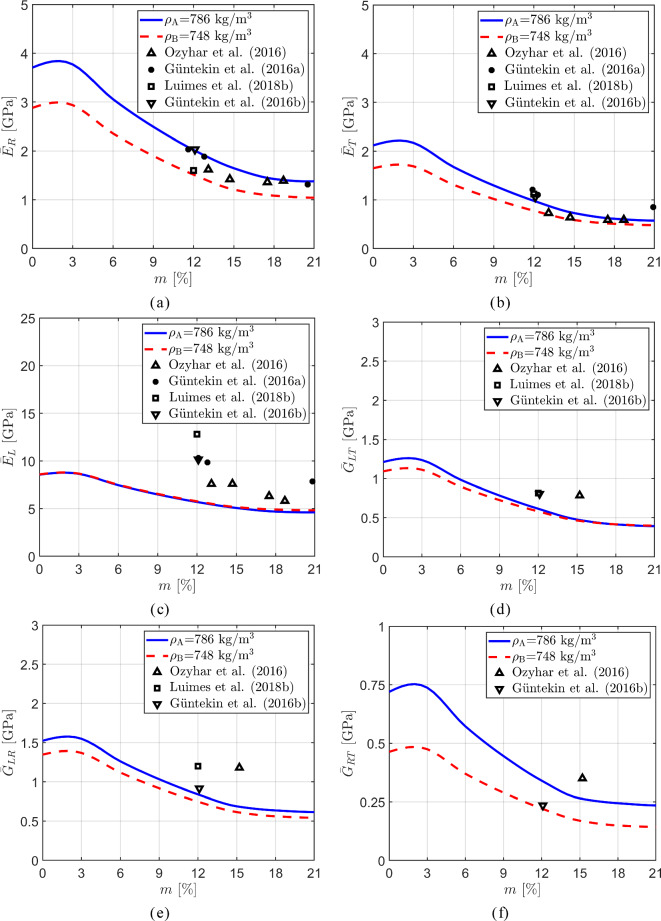
Macro-scale results: Macro-scale *Young’s moduli* and *shear moduli* of oak as a function of the moisture content. **a** Effective Young’s modulus $\bar{E}_{R}$ in the radial direction. **b** Effective Young’s modulus $\bar{E}_{T}$ in the tangential direction. **c** Effective Young’s modulus $\bar{E}_{L}$ in the longitudinal direction. **d** Effective shear modulus $\bar{G}_{LT}$ in the tangential plane. **e** Effective shear modulus $\bar{G}_{LR}$ in the radial plane. **f** Effective shear modulus $\bar{G}_{RT}$ in the transverse plane. The experimental data have been taken from [26, 27, 47, 54]

Figure 8 shows the Young’s moduli and shear moduli as a function of the moisture content $m$. Here, the blue solid and red dashed lines refer to the meso-scale unit cells A and B, with growth ring densities $\rho _\textrm{A}=786$ kg/m$^3$ and $\rho _\textrm{B}=748$ kg/m$^3$, respectively, whose meso-scale characteristics are listed in Table 1. Figure 8a–c respectively illustrate the effective Young’s moduli of oak in the radial direction, $\bar{E}_R$, tangential direction, $\bar{E}_T,$ and longitudinal direction, $\bar{E}_L$. The trends computed for $\bar{E}_R$ and $\bar{E}_T$ reveal that sample A in the radial and tangential directions is characterized by a higher stiffness than sample B, irrespective of the value of the moisture content. This feature may be ascribed to the fact that the effective density of sample A is 5% larger than that of sample B, and that the relatively dense latewood region of sample A has a 17% larger width ($W-W_e$) than that of sample B, see Table 1. Remarkably, the Young’s modulus $\bar{E}_L$ appears to be insensitive to these global and local differences in density, showing a trend that is almost identical for the two samples. In our previous work [43], however, it was observed that a higher material density of oak may lead to a higher Young’s modulus $E_L$, although it should be mentioned that the considered test samples then had a much larger difference in material density, on the order of 40%. Note further from Fig. 8a–c that all three Young’s moduli are characterized by a slightly increasing trend at a relatively small moisture content, followed by a monotonic decrease at higher moisture content values. The initial increase in the curves is due to the moisture-dependent behaviour of the cell walls illustrated in Fig. 4, which originates from the moisture-dependent response of lignin at the nano-scale, see [43] for more details on this aspect.

Figure 8a–c also depict the experimental data reported in [26, 27, 47, 54]. Despite some scatter, the experimental data are in relatively good agreement with the model predictions, showing a comparable decreasing trend under growing moisture content. The results further indicate that the Young’s modulus $\bar{E}_L$ in the longitudinal direction has the largest value, followed by $\bar{E}_R$ and finally $\bar{E}_T$. The fact that the stiffness $\bar{E}_R$ is larger than $\bar{E}_T$ can be attributed to the prevalent alignment and the denser configuration of fiber cells in the radial direction compared to the tangential direction, and the stiffening effect of the rays in the radial direction [58]. At the highest moisture content considered, ${m} = 21\%$, the ratio $\bar{E}_R/\bar{E}_T$ following from the computational results ranges between $2.59-2.71$, with the exact value depending on the material density. This range lies somewhat above the experimental value $\bar{E}_R/\bar{E}_T \approx 2$ reported in [12] for a *fully saturated* pedunculate oak wood sample with a dry density of 530 kg/m$^3$.

Figure 8d–f show the effective shear moduli of oak associated with the tangential plane $\bar{G}_{LT}$, the radial plane $\bar{G}_{LR}$, and the transverse plane $\bar{G}_{RT}$, as a function of the moisture content ${m}$, together with experimental data reported in the literature [27, 47, 54]. Similar to the Young’s moduli, the shear moduli increase with increasing material density $\rho$. Additionally, under a growing moisture content, they show a comparable trend to the Young’s moduli, which is characterized by a small initial increase, followed by a substantial monotonic decrease as the moisture content further increases.

Although not illustrated here, the effective Poisson’s ratios $\bar{\nu }_{LT}$, $\bar{\nu }_{LR}$ and $\bar{\nu }_{RT}$ characterizing the orthotropic oak wood only show a mild variation under an increasing moisture content, which is in line with experimental data [26, 27, 47, 54] and modelling results [43] reported in previous works. Accordingly, the visualization of the moisture-dependent response is omitted here for brevity. Instead, Table 3 summarizes the effective Poisson’s ratios of the meso-scale unit cells A and B at the reference moisture content $m = 12 \%$. Note that for each of the three Poisson’s ratios the values of the two samples differ only slightly and are in good agreement with the experimental and numerical values reported in the works mentioned above.

**Table 3:** Macro-scale results: Macro-scale *Poisson’s ratios* of oak, corresponding to a reference moisture content ${m} = 12\%$

Sample	Poisson’s ratio [-]		
	$\bar{\nu }_{LT}$	$\bar{\nu }_{LR}$	$\bar{\nu }_{RT}$
A ($\rho _\textrm{A}=786$ kg/m$^3$)	0.40	0.22	0.41
B ($\rho _\textrm{B}=748$ kg/m$^3$)	0.43	0.26	0.39

#### Hygro-expansion coefficients

**Fig. 9:**
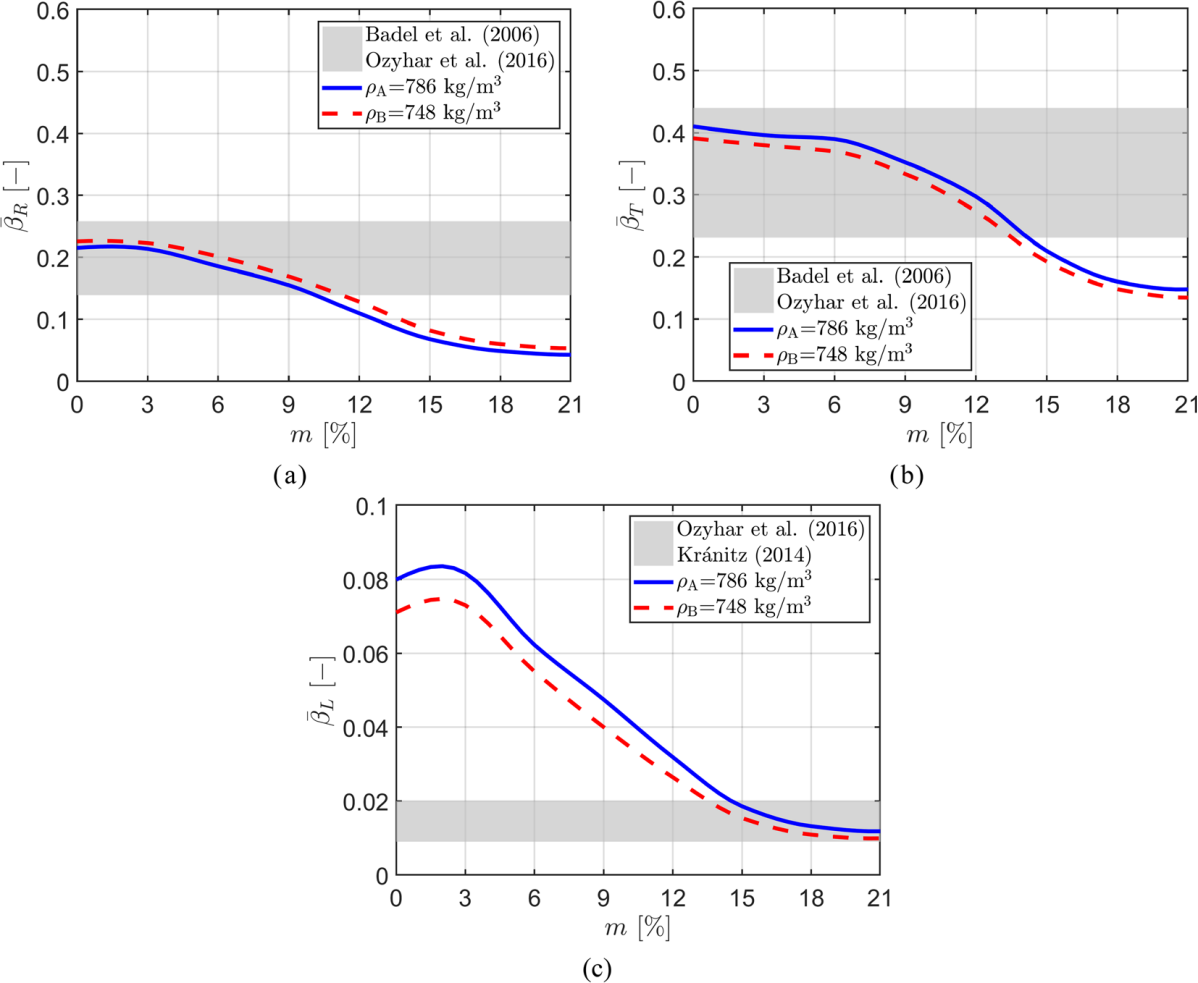
Macro-scale results: Macro-scale *hygro-expansive properties* of oak as a function of the moisture content. **a** Effective hygro-expansion coefficient $\bar{\beta }_{R}$ in the radial direction. **b** Effective hygro-expansion coefficient $\bar{\beta }_{T}$ in the tangential direction. **c** Effective hygro-expansion coefficient $\bar{\beta }_{L}$ in the longitudinal direction. The spread of the experimental data taken from [2, 38, 54] is indicated by the gray region

Defining the orthotropic macro-scale hygroscopic tensor of oak as

39 $$\begin{aligned} \begin{array}{lcl} \bar{\varvec{\beta }}= &   \bar{\beta }_{R} \textbf{e}_x\otimes \textbf{e}_x + \bar{\beta }_{T}\textbf{e}_y\otimes \textbf{e}_y + \bar{\beta }_{L}\textbf{e}_z\otimes \textbf{e}_z ~, ~\end{array} \end{aligned}$$

the three parameters $\bar{\beta }_{R}$, $\bar{\beta }_{T}$, and $\bar{\beta }_{L}$ represent the hygroscopic expansion coefficients in the radial, tangential, and longitudinal directions, respectively. These coefficients are derived directly from the diagonal components of the hygro-expansion tensor computed from equation (37)$_2$. Fig. 9 displays the effective hygroscopic coefficients of the oak samples as a function of the moisture content $m$. The blue solid and red dashed lines represent the meso-scale unit cells A and B, with densities $\rho _\textrm{A}=786$ kg/m$^3$, $\rho _\textrm{B}=748$ kg/m$^3$, respectively. The experimental results presented in Fig. 9 are based on the test data reported in [2, 38, 54]; since the moisture content values at which the hygro-expansion coefficients were measured are not reported in these references, only the spread in experimental data has been displayed by using a gray region of constant bandwidth. It is observed that the experimental bandwidths for the three hygroscopic coefficients mostly fall within the ranges of the modelling results and that a change in material density has a limited influence on the hygro-expansion coefficients. Additionally, the hygro-expansion coefficients typically decrease with an increase in moisture content, except for the longitudinal component $\bar{\beta }_{L}$, where a slight initial increase can be observed that is caused by the characteristic moisture-dependent behaviour of lignin [16, 43]. The hygro-expansion coefficient in the tangential direction, $\bar{\beta }_{T}$, is larger than in the radial direction, $\bar{\beta }_{R}$. Specifically, depending on the material density, the ratio $\bar{\beta }_{T}$/$\bar{\beta }_{R}$ ranges between $2.24-4.49$. This result corresponds reasonably well with experimental results for oak samples with an average density of 700 kg/m$^3$, for which $\bar{\beta }_{T}$/$\bar{\beta }_{R} \approx 1.78$, as reported in [2]. Finally, the hygro-expansion coefficient in the longitudinal direction is much smaller than in the other two directions, which is a typical feature of (oak) wood [38, 54].

### Local stress and strain fields

**Fig. 10:**
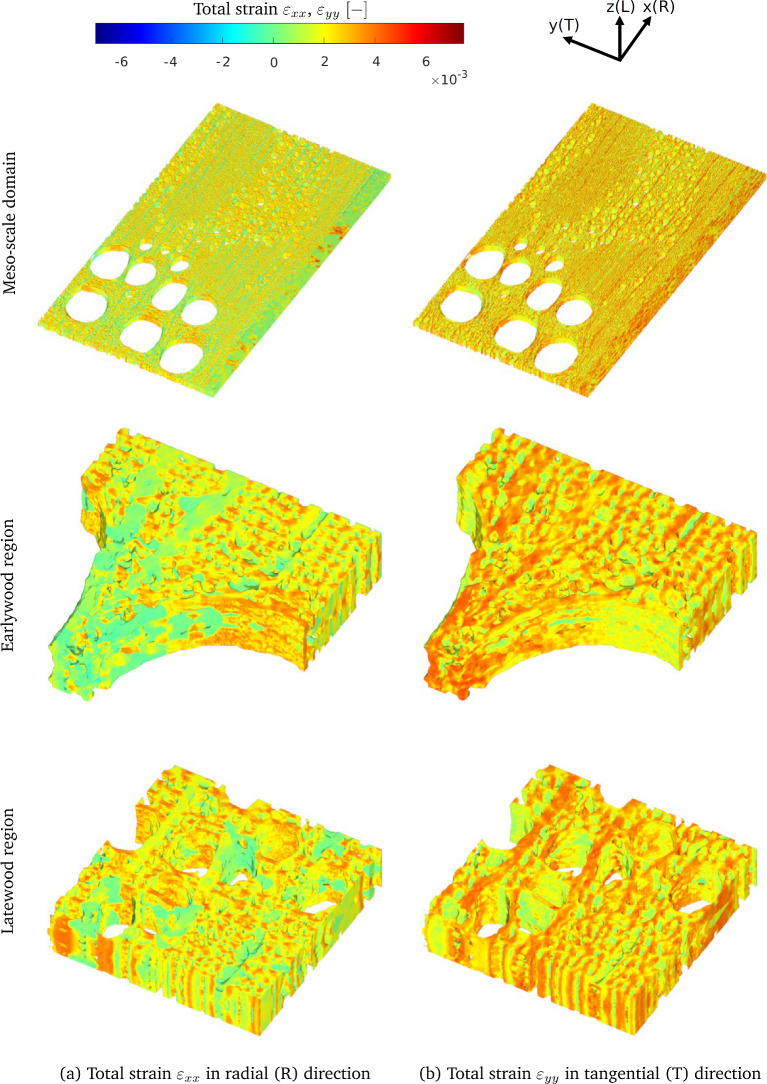
Meso-scale results: Meso-scale strain distributions in the oak growth ring (sample B) under *free expansion* conditions, computed for a moisture content variation $\Delta m = 1 \%$ with respect to a reference moisture content $m_0 = 12 \%$. **a** Total strain $\varepsilon _{xx}$ in radial (R) direction. **b** Total strain $\varepsilon _{yy}$ in tangential (T) direction. The results are depicted for the entire meso-scale domain (top row), and for detailed regions containing earlywood (middle row), and latewood (bottom row)

**Fig. 11:**
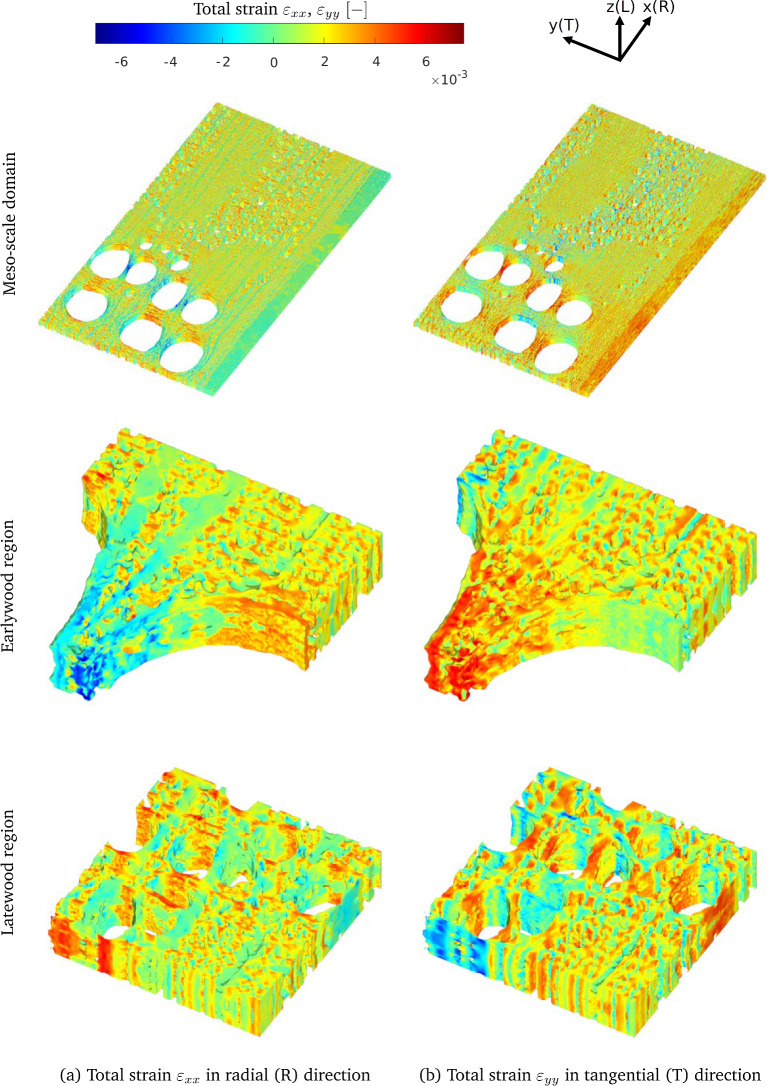
Meso-scale results: Meso-scale strain distributions in the oak growth ring (sample B) under *constrained expansion* conditions, computed for a moisture content variation $\Delta m = 1 \%$ with respect to a reference moisture content $m_0 = 12 \%$. **a** Total strain $\varepsilon _{xx}$ in radial (R) direction. **b** Total strain $\varepsilon _{yy}$ in tangential (T) direction. The results are depicted for the entire meso-scale domain (top row), and for detailed regions containing earlywood (middle row), and latewood (bottom row)

With the present multi-scale model, the total strain and stress fields in a meso-scale unit cell can be computed using equations (6) and (7), respectively. Correspondingly, Figs. 10 and 11 illustrate the distributions of the total mesoscopic strain in sample B under, respectively, the *free expansion* condition and *constrained expansion* condition defined by equation (6), as computed for a moisture content variation $\Delta m = 1 \%$ with respect to a reference moisture content $m_0 = 12 \%$. Using the sorption isotherms for oak wood reported in [45], this variation in moisture content translates into a change in relative humidity from RH=56% to 61% along the boundary curve for desorption, and from RH=75% to 79% along the boundary curve for adsorption, respectively. Here, it is noted that a change in relative humidity of $\Delta$RH $\approx 5\%$ is in agreement with the maximal allowable relative humidity variation adopted in large international museums for the preservation of their art collections [46].

**Fig. 12:**
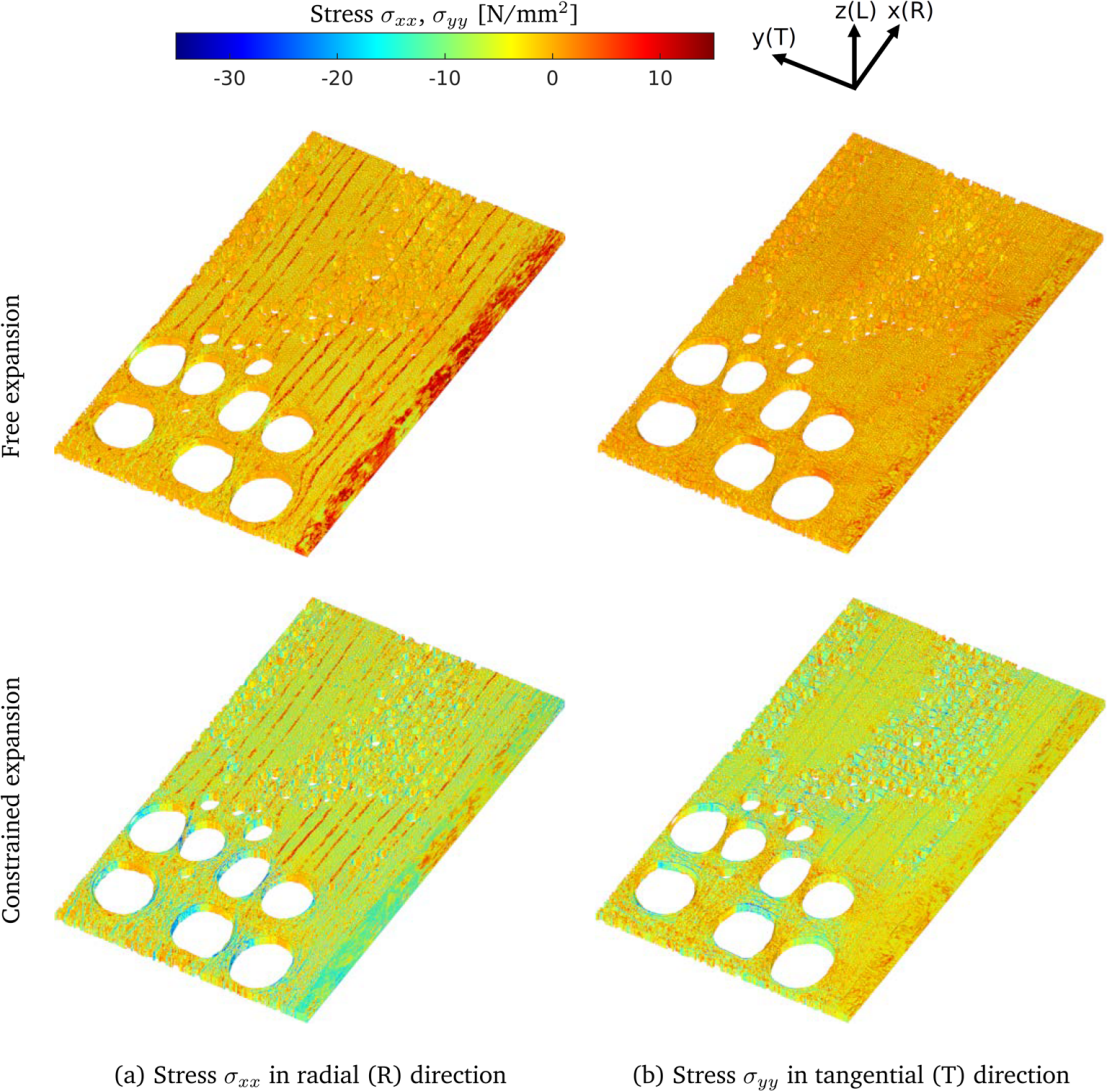
Meso-scale results: Meso-scale stress distributions in the oak growth ring (sample B) under *free expansion* (top row) and *constrained expansion* (bottom row) conditions, as computed for a moisture content variation $\Delta m = 1 \%$ with respect to a reference moisture content $m_0 = 12 \%$. **a** Stress $\sigma _{xx}$ in radial (R) direction. **b** Stress $\sigma _{yy}$ in tangential (T) direction

In Figs. 10 and 11, the strain components $\varepsilon _{xx}$ in radial (R) direction and $\varepsilon _{yy}$ in tangential (T) direction are depicted for the entire meso-scale domain (top row), and further in detailed regions containing earlywood (middle row) and latewood (bottom row). The corresponding stress components $\sigma _{xx}$ and $\sigma _{yy}$ in the radial and tangential directions are illustrated in Fig. 12a, b, respectively, under free expansion (top row) and constrained expansion (bottom row). The strain component $\varepsilon _{zz}$ in the longitudinal (L) direction has not been depicted, since the hygroscopic deformation computed in this direction is significantly smaller than in the radial and tangential directions, which can be confirmed from the differences in the values of the hygro-expansion coefficients depicted in Fig. 9.

Under free expansion conditions, the average macroscopic deformation shown in Fig. 10 is purely hygroscopic. The mesoscopic strain $\varepsilon _{xx}$ in the radial direction (Fig. 10a) is, on average, smaller than the strain $\varepsilon _{yy}$ in the tangential direction (Fig. 10b). This is, because the hygro-expansion coefficient in the radial direction is smaller than in the tangential direction, $\bar{\beta }_R < \bar{\beta }_T$, see Fig. 9. Observe, however, that both strain distributions are characterized by substantial fluctuations around the average hygroscopic strain imposed from the macro-scale. These fluctuations are caused by local hygro-mechanical interactions arising from inhomogeneities in morphology and cell wall material properties. Additionally, the band-shaped multi-seriate and uni-seriate ray regions oriented in the radial (R) direction experience the largest tensile strain, as indicated by the thin, red-coloured strips in the contour plot of Fig. 10 (top row). It can be further confirmed from Fig. 12 (top row) that the stresses $\sigma _{xx}$ and $\sigma _{yy}$ generated under free expansion are on average zero. Nevertheless, the mesoscopic stresses may locally reach tensile values up to 12 N/mm$^2$, as, for example, occurs in the red-coloured, band-shaped ray region located near the right edge of the sample, see Fig. 12a—top row. Despite that these stress values are well within the range of macro-scale tensile strengths of $4-22$ N/mm$^2$ measured for historic oak wood (dated 1300 A.D. and 1668 A.D.) in the direction perpendicular to the grain, see Figure 20 in [47], it is anticipated that they will not lead to damage, since the meso-scale tensile strength of individual cell walls may be expected to lie above this range. It is important, however, to accurately validate this hypothesis, because the growth and coalescence of meso-scale damage sites can result in undesirable cracks and deformations at the macro-scale, as occasionally observed in ancient, susceptible oak museum objects (i.e., historical oak panel paintings and oak cabinets) exposed to ambient indoor climate fluctuations [72–74]. Hence, additional experiments are required at the meso-scale, in which the tensile strength of (ray) cell walls present in oak samples taken from historical museum objects is measured, and subsequently compared against the mesoscopic tensile stresses computed with the current multi-scale model. In addition to the stress patterns presented in Fig. 12, the meso-scale stress fields generated under alternative, more critical hygro-mechanical conditions representative of oak museum objects can then also be analysed. The outcome of these analyses will reveal whether a relative humidity fluctuation of $\Delta$RH=5% (or possibly higher) may be adopted by international museums as an unconditional, safe margin for the preservation of their historical oak objects. Finally, note that the assumption of a uniform mesoscopic moisture content adopted in the current modelling approach is consistent with the relatively stable hygro-mechanical loading typically observed in museum environments. However, even under museum conditions, local moisture gradients may occasionally develop due to rapid temporal changes in relative humidity [51]. The simulation of such scenarios requires the development of a homogenization framework that includes the mesoscale interactions between moisture transport and the mechanical response, which may be a topic for future work.

Under constrained expansion, the average macroscopic deformation corresponding to the mesoscopic strain distributions in Fig. 11 is equal to zero, which thus defines the strain level about which the mesoscopic strains fluctuate. Observe that the highest tensile strains appear around the earlywood vessels and in the multi-seriate ray region along the right specimen edge. In contrast to the case of free expansion, both the earlywood and latewood regions also experience compressive strains (indicated in blue), compare Figs. 11 and 10 (middle row and bottom row). In addition, the stresses developing in the meso-structure on average are compressive, see Fig. 12 (bottom row), and in regions adjacent to the vessels reach an extreme value of approximately -25 N/mm$^2$, both in the radial and tangential direction. These regions may thus be prone to micro-buckling, which is a failure mechanism often observed in wood cell walls [18, 25]. Further, in some parts of the multi-seriate and uni-seriate ray regions relatively high tensile stresses occur in the radial direction, up to a value of 12 N/mm$^2$. However, as the comparison of Fig. 12 (bottom row) with Fig. 12 (top row) shows, the areas with high tensile stresses are smaller than under free expansion conditions.

## Conclusions

A computational multi-scale model is presented for the prediction of the macroscopic hygro-mechanical behaviour of oak wood, which is based on including detailed, three-dimensional mesoscopic representations of entire oak growth rings, obtained by X-ray micro-computed tomography ($\mu$CT). The 3D meso-structural volume acquired by $\mu$CT scanning consists of an array of voxels, with the grayscale intensity values of the voxels denoting the local material densities. A level set-based image segmentation method is applied to these data to distinguish the individual meso-structural phases, including the cell walls and voids (lumen and vessels). A dedicated algorithm based on the spatial gradient of the level set function is developed to accurately identify the local material directions in the cell walls. The individual phases in the meso-scale cellular structure are discretized using the extended finite element method. Here, a so-called moment fitting scheme is applied for the efficient numerical integration of the hygro-mechanical response in the elements intersected by cell wall boundaries. Finally, asymptotic homogenization is used for computing the effective response of oak wood at the macro-scale from the hygro-mechanical response of the underlying meso-structure.

The macro-scale hygro-mechanical behaviour calculated for the oak growth rings is orthotropic, and the constitutive parameters generally agree well with experimental values reported in the literature. The elastic and hygro-expansion coefficients show a decrease under increasing moisture content, and the elastic response becomes stiffer for a higher material density. Further, the meso-scale response computed for oak growth rings subjected to a representative moisture content variation allows to accurately identify local critical sites characterized by a stress level at which mesoscopic hygro-mechanical damage may occur. The effective mechanical and hygroscopic properties calculated by the multi-scale model may serve as input for the prediction of the moisture-dependent mechanical response of oak wood structures and objects subjected to arbitrary hygro-mechanical loading paths.

To further investigate the phenomena discussed in this paper, more systematic experimental studies on oak wood are necessary, especially on the identification of the critical hygro-mechanical conditions for nucleation and propagation of damage mechanisms at the mesoscopic scale and the scales below. The sensitivity of the results to the relatively large variety in meso-scale morphology of oak wood is also a topic for future study.

## Footnotes

1 The hygro-mechanical properties in equations (11)$_{1,2}$ are defined with respect to a local (1, 2, 3) Cartesian coordinate system directed along the principal material directions of the cell walls, with the $1-$axis oriented parallel to the cell wall, the $2-$axis oriented perpendicular to the cell wall, and the $3-$axis in the longitudinal direction of the cell wall.
